# A chromosome‐level genome assembly provides insights into ascorbic acid accumulation and fruit softening in guava (*Psidium guajava*)

**DOI:** 10.1111/pbi.13498

**Published:** 2020-11-12

**Authors:** Chen Feng, Chao Feng, Xinggu Lin, Shenghui Liu, Yingzhi Li, Ming Kang

**Affiliations:** ^1^ Key Laboratory of Plant Resources Conservation and Sustainable Utilization South China Botanical Garden Chinese Academy of Sciences Guangzhou China; ^2^ University of Chinese Academy of Sciences Beijing China; ^3^ South Subtropical Crops Research Institute Chinese Academy of Tropical Agriculture Sciences Zhanjiang China; ^4^ Horticulture and Forestry Department Guangdong Ocean University Zhanjiang China; ^5^ Innovation Academy of South China Sea Ecology and Environmental Engineering Chinese Academy of Sciences Guangzhou China

**Keywords:** Myrtaceae, ascorbic acid, starch degradation, fleshy fruit, PacBio sequencing, Hi‐C

## Abstract

Guava (*Psidium guajava*) is an important fleshy‐fruited tree of the Myrtaceae family that is widely cultivated in tropical and subtropical areas of the world and has attracted considerable attention for the richness of ascorbic acid in its fruits. However, studies on the evolution and genetic breeding potential of guava are hindered by the lack of a reference genome. Here, we present a chromosome‐level genomic assembly of guava using PacBio sequencing and Hi‐C technology. We found that the genome assembly size was 443.8 Mb with a contig N50 of ~15.8 Mb. We annotated a total of 25 601 genes and 193.2 Mb of repetitive sequences for this genome. Comparative genomic analysis revealed that guava has undergone a recent whole‐genome duplication (WGD) event shared by all species in Myrtaceae. In addition, through metabolic analysis, we determined that the L‐galactose pathway plays a major role in ascorbic acid biosynthesis in guava fruits. Moreover, the softening of fruits of guava may result from both starch and cell wall degradation according to analyses of gene expression profiles and positively selected genes. Our data provide a foundational resource to support molecular breeding of guava and represent new insights into the evolution of soft, fleshy fruits in Myrtaceae.

## Introduction

Guava, *Psidium guajava*, is among the many economically important plant species in the plant family Myrtaceae, which also includes eucalyptus (*Eucalyptus grandis*), wax apple (*Syzygium samarangense*) and rose myrtle (*Rhodomyrtus tomentosa*; Grattapaglia *et al*., 2012; Wilson *et al*., 2004). *Psidium guajava* was described by Linnaeus in 1753 based on collections from Asia, but many botanists consider the species to be native to tropical America, probably occurring naturally from southern Mexico to South America, with subsequent introductions to many other tropical and subtropical regions throughout the world over the past 400 years (Cobley, 1976; Morton, 1987; Samson, 1986). Guava has been designated as 'the apple of the tropics' or 'poor man's fruit' because it thrives on a variety of soils, propagates easily and bears fruit relatively quickly (Nakasone and Paull, 1998; Singh, 2007). Among ~3500 fleshy‐fruited plants in Myrtaceae (Biffin *et al*., 2010), the fruit of guava is the only commercially significant one. Its commercial success may largely result from the facts that it is cheap to grow and purchase and is an extremely rich source of ascorbic acid (i.e. vitamin C) and dietary fibres, as well as a good source for vitamins A and B_2_ and various other minerals (Gutierrez *et al*., 2008; Rahman *et al*., 2003). The fruit is also reported to possess many pharmacological properties (Gutierrez *et al*., 2008). Guava is consumed as fresh or dried fruits, jam, and antioxidant additives and is important in local markets and as an international commodity in over 60 tropical and subtropical countries (CABI, 2019). Guava is most widely produced in Central and South America (Brazil, Mexico), India, and Thailand, and has steadily increasing production in the Caribbean, Hawaii and Florida (USA), and South Africa (CABI, 2019). According to Food and Agriculture Organization (FAO) of the United Nations, the average annual production of guava is ca. 6.8 million tons since 2017 (FAO, 2018).

Ascorbic acid is essential for human health because it is required for immune system function (Sorice *et al*., 2014) and many physiological processes such as repair of connective tissues, collagen synthesis and producing neurotransmitters (reviewed in Grosso *et al*., 2013). Ascorbic acid can be metabolized by most mammals except humans due to the mutation of the *L‐gulono‐galactone oxidase* (*GLO*) gene that occurred ~61 million years ago (Drouin *et al*., 2011). Therefore, dietary ascorbate is an indispensable nutrient for humans. Due to human needs for dietary sources of ascorbic acid, considerable efforts have been made to boost the accumulation of ascorbic acid in plants using genes encoding enzymes in biosynthesis and regenerating pathways (reviewed in Macknight *et al*., 2017). Four *de novo* biosynthesis pathways of ascorbic acid in plants have been proposed: the L‐galactose, L‐gulose, *myo*‐inositol and D‐galacturonate pathways (Broad *et al*., 2020; Ishikawa *et al*., 2018; Yoshimura and Ishikawa, 2017). Notably, guava fruits produce sixfold more ascorbic acid than strawberry and fivefold more than kiwifruit or orange (Davey *et al*., 2000; Gutierrez *et al*., 2008; Kumrawat, 2018), and the extracts from guava fruits and leaves are potential sources of many other natural antioxidants such as anthocyanin, lycopene, phenolics and tannins (Fernandes *et al*., 2014; Guevara *et al*., 2019; Yang *et al*., 2007). Nevertheless, the genetic basis for ascorbic acid and other bioactive compounds in guava have rarely been studied, except for development of several molecular markers associated with ascorbic acid (Youssef and Ibrahim, 2016). Major obstacles to the improvement of ascorbic acid and other bioactive compounds in guava using molecular breeding are the absence of reference genome sequences and transcriptomic information.

Plants have evolved different types of fruits to protect or disperse their seeds. For example, capsular fruits generate dry, hard pericarps via lignification (reviewed in Dardick and Callahan, 2014) and disperse seeds by physical forces such as wind, water or adhering to animal surfaces (Scutt *et al*., 2006). In contrast, fleshy fruits often surround their seeds with soft edible tissues (reviewed in Dardick and Callahan, 2014) so that they are dispersed by animal consumption and excretion. Evolution of fruit types has attracted the attention of many botanists and others over the past decade (Bobrov and Romanov, 2019; Ho and Costea, 2018; Pabón‐Mora and Litt, 2011). The hardening process of woody‐capsular fruits is thought to be similar to wood formation in xylem, which has been well studied in model plants and crop species (Dardick and Callahan, 2014). However, mechanisms underlying fleshy fruit softening are still not well understood, due, in part, to their multiple independent evolutionary origins (Knapp, 2002; Li *et al*., 2019).

Fruits of Myrtaceae are classified as either fleshy or capsular, and fruits comprise the traditional basis for taxonomic division of the family into two subfamilies: fleshy‐fruited Myrtoideae and capsular‐fruited Leptospermoideae (Niedenzu, 1893). However, molecular phylogenetic studies show that the fleshy‐fruited species are not a monophyletic group and that fleshy fruits have evolved independently several times in the family (Biffin *et al*., 2010; Sytsma *et al*., 2004; Thornhill *et al*., 2015). This makes Myrtaceae an ideal system to study fruit softening mechanisms and evolution of fruit types. Previous studies in guava showed that four enzymes involved in cell wall degradation and had low activity in mature fruits or only slightly elevated activity during ripening (Ali *et al*., 2004). Moreover, Braga *et al*. (2017) found that the amount of pectin in cell walls in guava fruits was too high during fruit maturation for its degradation to explain the softening behaviour. Thus, fruit ripening in guava may follow the starch degradation model. However, most studies of fruit softening mechanisms in Myrtaceae have primarily focused on enzymatic activity, while the genetic mechanisms of fruit softening in the family during fleshy fruit formation have not been reported. Therefore, a genome assembly of guava will provide a reference for the understanding of softening mechanisms. In addition, comparison of genomic information for a fleshy fruit (*P. guajava*) and representative woody‐capsular‐fruited plants, *E. grandis* and *Leptospermum scoparium* (mānuka), will provide a new sight in evolution of fruit types in Myrtaceae.

Here, we assembled a chromosome‐level genome for guava using third‐generation PacBio sequencing and Hi‐C techniques. We determined the major route to ascorbic acid accumulation in guava was the L‐galactose pathway. Comparative genomic analyses revealed the importance of starch degradation in fleshy fruit formation in Myrtaceae. Our genomic assembly of guava represents a foundation for investigating the origins of fleshy fruits in Myrtaceae and for accelerating genetic improvement of guava.

## Results

### Genome sequencing and assembly

**Figure 1:**
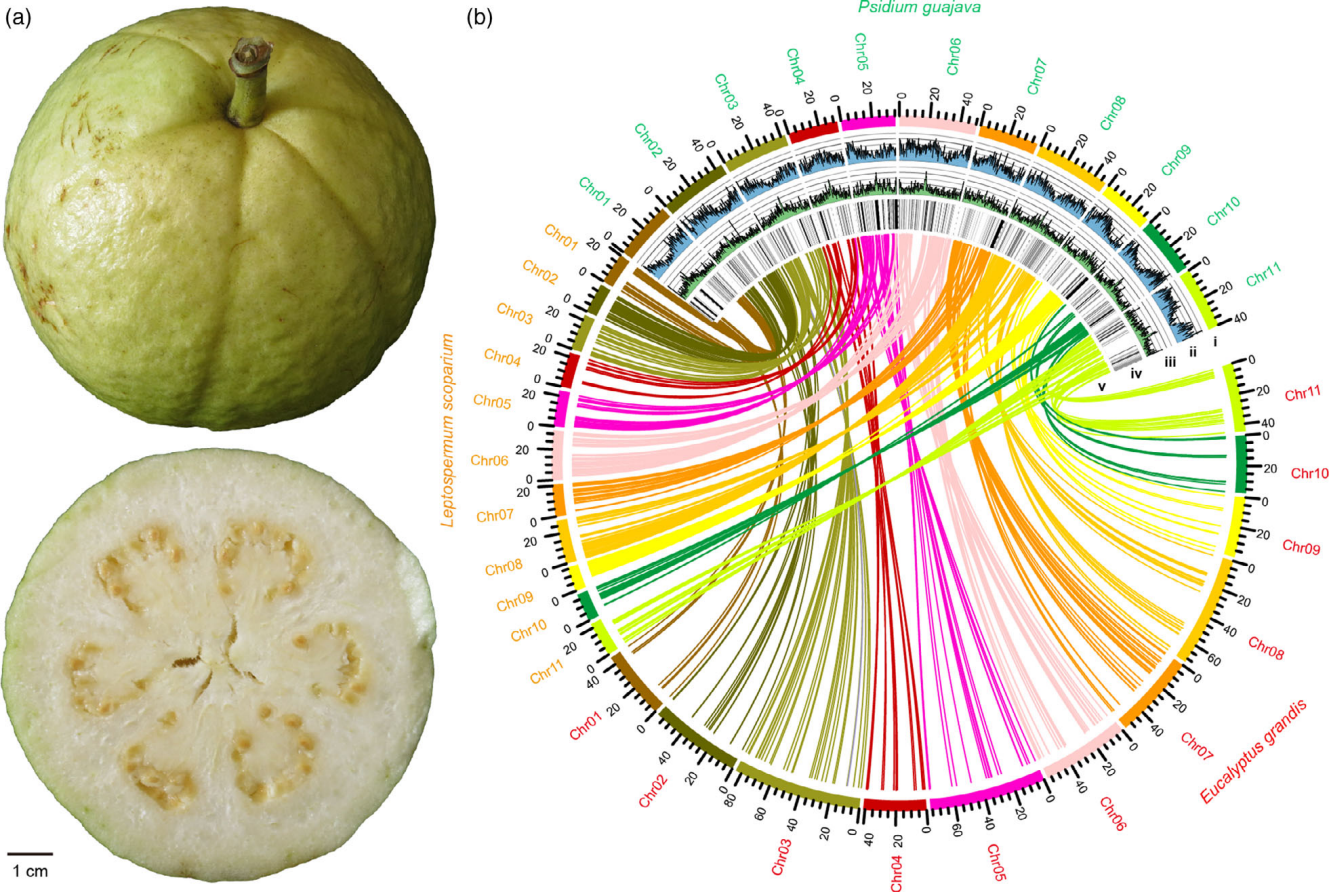
(a) Representative fruit from ‘New Age’ cultivar of guava sequenced in this study; (b) circos view of the *P. guajava*, *E. grandis* and *L. scoparium* genomes. Lanes depict circular representation of pseudochromosomes (i) and the density of genes, transposons and tandem duplicates (ii‐iv). Lines (v) in the inner circle represent syntenic relationships between the genomes of *P. guajava* and *E. grandis*, and *L. scoparium*.

We sequenced the genome of the guava cultivar, ‘New Age’ (2*n* = 2*x* = 22) (Figure 1a), which is commonly grown in Guangdong Province, China. We sequenced it using a combination of short‐read sequencing from Illumina NovaSeq with the PE 150 bp protocol and SMRT from PacBio Sequel platform. The sequencing generated ~53.2 Gb of high‐quality short‐read sequences and ~53.6 Gb of PacBio sequences, both representing over 115× coverage for the genome, which has an estimated size of 463.8 Mb based on the 17‐mer depth distribution analysis of the sequenced reads (Figure S1) and previous flow cytometry analysis by Coser *et al*. (2012). The final assembled sequence was 443.8 Mb, representing 95.7% of the guava genome. The assembly consisted of 44 scaffolds with an N50 of 40.4 Mb and 73 contigs with an N50 of 15.8 Mb (Table 1; Table S1). We used the Hi‐C technology to reorder and anchor the total 99.44 % (441.27 Mb) of the genome onto 11 pseudochromosomes (Figure S2). The GC content of the assembled guava genome was 39.5%, which is similar to those of *E. grandis* (Myburg *et al*., 2014) and *L. scoparium* (Thrimawithana *et al*., 2019), the two most closely related species to guava with sequenced genomes.

**Table 1:** Summary statistics of the genome assembly of guava and annotations

Feature	Value
Estimated genome size (Mb)	463.8
Total size of assembled scaffold (Mb)	443.76
Scaffold N50 (Mb)	40.4
Longest scaffold (Mb)	50.6
Total size of assembled contigs (Mb)	443.75
Number of contigs (≥1 kb)	73
N50 contig length (Mb)	15.8
Largest contig (Mb)	37.0
GC content (%)	39.5
Number of gene models	25 601

We used BWA v0.7.17 (Li, 2014) to align Illumina short reads to the assembly, and obtained the mapping rate of 96.75%. We then assessed the quality of the guava genome assembly through the Conserved Core Eukaryotic Gene Mapping Approach (CEGMA) (Parra *et al*., 2007). The analysis revealed that 94.4% of the core protein‐coding genes were recovered in our assembled genome (Table S2). In addition, we found 1352 (93.9%) complete gene models and 28 (1.9%) fragmented gene models out of 1440 Benchmarking Universal Single‐Copy Orthologs (BUSCO) genes (Simão *et al*., 2015; Table S3). Furthermore, 97.7% of expressed sequence tags (ESTs) were covered by the assembly, with >50% sequence identity (Table S4). Moreover, we successfully mapped 91.6%–94.1% of RNA‐Seq datasets generated from different tissues and developmental stages to the assembled genome of guava (Table S5). Taken together, these results suggest a high‐quality genome assembly of guava.

### Repeat annotation and gene prediction

We analysed repetitive sequences by combining *de novo* prediction and a homology‐based search at both the DNA and protein levels, and found that repetitive sequences occupy 43.55% (193.2 Mb) of the genome of guava (Figure 1b; Table S6). We performed an annotation pipeline combining *de novo*, homolog‐based search and RNA‐Seq methods to predict gene models from the repeat‐masked guava genome sequence (Table S5; Figure S3). These analyses predicted a total of 25 601 protein‐coding genes, representing 23.56% of the genome assembly (Table S7). This is a much smaller number of genes than in the sequenced genomes of the myrtaceous species, *L. scoparium* and *E. grandis* (Myburg *et al*., 2014; Thrimawithana *et al*., 2019). Among the 25 601 genes, 25 428 (99.3%) were functionally annotated (Table S8). There were 25 498 genes (99.59%) present on chromosomally anchored contigs. In a Gene Ontology (GO) analysis, 9724 (37.98%), 12 866 (50.26%) and 13 400 (52.3%) of annotated genes were assigned to the GO terms cellular component, molecular function and biological process, respectively (Figure S4). In addition to the protein‐coding genes, we also identified 330 miRNA genes, 405 tRNA genes and 844 rRNA genes in the guava genome (Table S9; Figure S5a). We also detected a total of 22 334 simple sequence repeats (SSRs) across the whole genome (Figure S5b).

### Gene family analysis

Compared with ten other genomes of angiosperms, we found a total of 4310 species‐specific single‐copy genes in guava (Tables S10 and S11). We conducted an analysis of GO annotations for these genes and found they were enriched in GO terms including regulation of cell death, receptor regulator activity and chromatin (Table S12).

**Figure 2:**
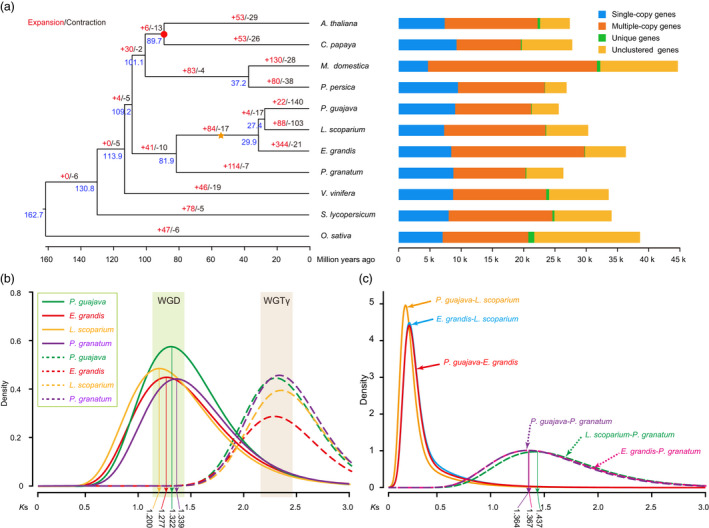
Phylogenetic relationship and comparative genomics analyses. (a) Left, phylogenetic tree of 11 plant species and evolution of gene families. Blue numerical value beside each node shows the estimated divergence time (MYA, million years ago). Red circle indicates the node age calibration point, and yellow star represents the inferred whole‐genome duplication event. Right, the distribution of single‐copy, multiple‐copy, unique and unclustered genes in 11 plant species. Distribution of synonymous substitution levels (*K*s) of syntenic orthologous (b) and paralogous genes (c).

The shared gene families among guava and ten other angiosperm species are summarized in Table S11. We found that guava shared more gene families with *L. scoparium* and *E. grandis* than with *Punica granatum* (pomegranate; Myrtales, Lythraceae; Figure S6). In total, we found that 22 gene families were expanded in guava, while 140 gene families experienced losses compared to the most recent common ancestor (MRCA) of guava and *L. scoparium* (Figure 2a). Guava showed fewer gene family expansions and more gene family contractions than the other species in Myrtaceae (Figure 2a), and this is consistent with the smaller number of genes we detected in guava compared to the other myrtaceous species. The expanded gene families are enriched in biological processes, especially stimulus response and phosphorelay signal transduction and in several molecular functions, particularly ADP binding and ribosyltransferase activity (Figures S7 and S8; Table S13). Functional analysis of contracted gene families indicated enrichment of GO terms such as response to auxin and terpene synthase activity (Figures S9 and S10; Table S14), suggesting possible function losses in the xylem formation (Soler *et al*., 2015) and essential oil producing (Myburg *et al*., 2014) pathways.

We examined the evolutionary relationships among guava and ten other plant species with sequenced genomes using phylogenetic analysis. The phylogenetic analysis, based on alignments of 487 single‐copy genes, supported the placement of *P. guajava*, *E. grandis*, and *L. scoparium* in Myrtaceae and *P. granatum* in the Myrtales order (Figure 2a). Interestingly, *P. guajava* and *L. scoparium* comprised a monophyletic group, which is inconsistent with phylogenetic analyses reported by Biffin *et al*. (2010) and Thornhill *et al*. (2015). To improve phylogenetic resolution among species of Myrtaceae, we reconstructed species trees using protein and DNA sequences of 3454 single‐copy orthologs with *P. granatum* as the outgroup. In these analyses, both the DNA and protein sequences yielded the topology of (*P. guajava*, *L. scoparium*, *E. grandis*, *P. granatum*) with high support (bootstrap values = 100%; Figure S11).

### Whole‐genome duplication

**Figure 3:**
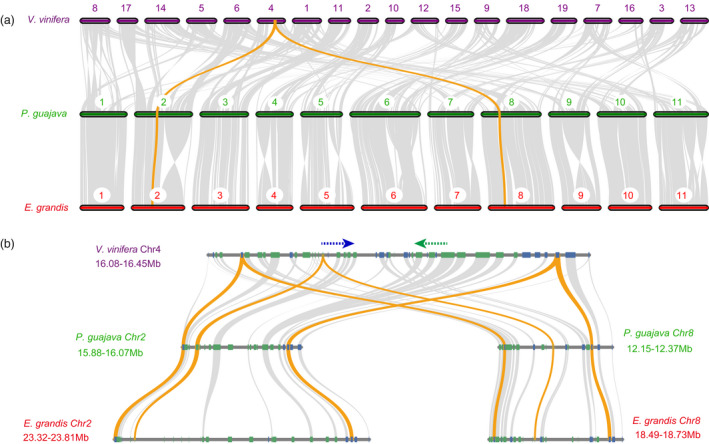
Collinearity patterns between *V. vinifera*, *P. guajava* and *E. grandis*. (a) Typical macro‐collinearity patterns between genomic regions from *V. vinifera*, *P. guajava* and *E. grandis*. The macro‐collinearity pattern shows that a typical ancestral region in the*V. vinifera* genome can be traced to two regions each in *P. guajava* and *E. grandis*; (b) Micro‐collinearity pattern between *V. vinifera*, *P. guajava* and *E. grandis*.

Distributions of synonymous substitutions per synonymous site (*K*s) for paralogous genes of guava showed a peak at *K*s ≈ 1.3 (Figure 2b; Figure S12), and similar peaks were identified in *L. scoparium* and *E. grandis* (Figure 2b; Figure S12). In addition, collinearity patterns between *Vitis vinifera* L. (common wine grape; Vitales, Vitaceae), *P. guajava*, and *E. grandis* (Figure 3) and synteny analyses among species of Myrtaceae (Figure 1; Figure S13) indicated that Myrtaceae underwent a whole‐genome duplication (WGD) event after the well‐known paleo‐hexaploidization event, γ, in the most recent common ancestor (MRCA) of all eudicots (Jiao *et al*., 2012; Myburg *et al*., 2014).

Qin *et al*. (2017) inferred that the MRCA of Myrtales underwent a WGD event after the γ event based on analysis of synteny patterns between *E. grandis* and *P. granatum*. However, the *K*s distributions for paralogous genes in our analyses revealed a little higher peak value in *P. granatum* than species of Myrtaceae (Figure 2b; Figure S12). Peak values of the *K*s distributions for orthologous genes between Myrtaceae and Lythraceae were a little larger than those within Myrtaceae (Figure 2b, c; Figure S12). However, differences in the distribution of *K*s between Myrtaceae and Lythraceae, represented by *P. granatum*, were too small to determine if they underwent a shared WGD event or independent ones. In order to precisely locate the phylogenetic position of WGDs in Myrtales, we conducted a Multi‐tAxon Paleopolyploidy Search (MAPS) analysis (Li *et al*., 2015) with seven species: *P. guajava*, *L. scoparium*, *E. grandis*, *P. granatum* of Myrtales and *Arabidopsis thaliana*, *V. vinifera*, and *Solanum lycopersicum* of Brassicales, Vitales and Solanales, respectively. We recovered 8587 phylogenies of gene families with at least one gene copy from each taxon. Based on MAPS, we identified 7572 trees representing these gene families that included the MRCA of Myrtaceae, and among these, 60% showed a WGD for the MRCA of Myrtaceae (Figure S14; Table S15). In contrast, only 5.8% supported a gene duplication shared between Myrtaceae and *P. granatum* (i.e. for Myrtales). (Figure S14; Table S15). These results support that WGD events in Myrtaceae occurred separately from those in Lythraceae, which is consistent with the finding of inferring putative ancient whole‐genome duplications in the 1000 Plants (Li and Barker, 2020; One Thousand Plant Transcriptomes Initiative, 2019).

We further determined the age of WGD events in Myrtales plants according to their distributions of *K*s (Badouin *et al*., 2017; Vanneste *et al*., 2013). Synonymous substitutions are putatively evolutionarily neutral and accumulate changes at a constant rate, which can be used to infer the age of WGDs. We inferred an average *K*s/year rate of 1.14 × 10^−8^ in Myrtales. Based on this, we inferred that the independent WGD events in Myrtaceae and Lythraceae occurred 50.1–61.2 million years ago (MYA) and 55.8–61.7 MYA with a confidence interval of 95%. Collectively, our results indicate that the WGD events in Myrtaceae and Lythraceae are family‐specific but occurred within similar timeframes.

### Ascorbic acid metabolism

**Figure 4:**
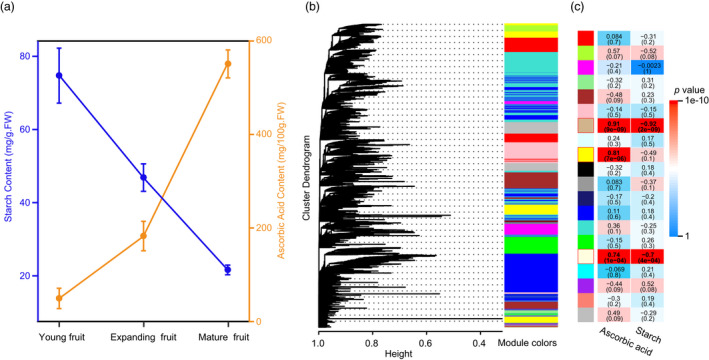
(a) Starch and ascorbic acid accumulation in fruits at various developmental stages of *P. guajava*; (b) WGCNA dendrogram indicating the expression of different gene modules in all 21 samples of *P. guajava*; and (c) analysis of relationships between traits and modules. Different colours represent different modules.

**Figure 5:**
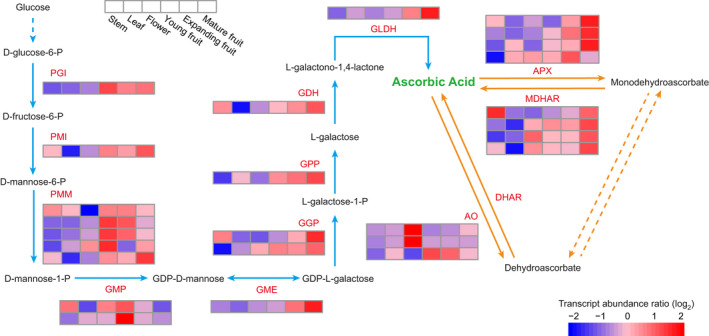
Transcript abundance of genes involved in ascorbic acid metabolism in guava. Blue arrows indicate the biosynthesis pathway, yellow arrows indicate the regeneration pathway, and arrows with dashed lines indicate undefined processes. Scaled log_2_ expression values (FPKM) are shown in the heat map legend. The six boxes in one row of each heat map (left to right) correspond to the expression levels in stem, leaf, flower and at three fruit developmental stages. Each row in the heat map corresponds to one gene. AO: L‐ascorbate oxidase; APX: L‐ascorbate peroxidase; DHAR: dehydroascorbate reductase; GDH: L‐galactose dehydrogenase; GGP: GDP‐L‐galactose phosphorylase; GLDH: L‐galactono‐1,4‐lactone dehydrogenase; GME: GDP‐D‐mannose‐3,5‐epimerase; GMP: GDP‐D‐mannose pyrophosphorylase; GPP: L‐galactose‐1‐phosphate phosphatase; MDHAR: monodehydroascorbate reductase; PGI: glucose‐6‐phosphate isomerase; PMI: mannose‐6‐phosphate isomerase; PMM: phosphomannomutase.

We measured ascorbic acid content in three different fruit developmental stages (young, expanding and mature). We found that ascorbic acid content increased along with fruit development (Figure 4a; Table S16). We investigated genes encoding key enzymes involved in all four known ascorbic acid biosynthesis pathways and the regeneration pathway (Figure S15) during guava fruit development. All genes associated with the L‐galactose pathway were found in guava genome, but Alase (aldonolactonase), which produces precursors of ascorbic acid in the galacturonate pathway, GlcUR (D‐galacturonate reductase) in the *myo*‐Inositol pathway, and a series of genes in the L‐gulose pathway were absent (Figure S15; Table S17). Comparisons with two other sequenced species of Myrtales, *E. grandis* and *P. granatum*, and with *A. thaliana* revealed no expansion of genes in the ascorbic acid biosynthesis pathways, but gene families responsible for regenerating ascorbic acid [AO (L‐ascorbate oxidase) and MDHAR (monodehydroascorbate reductase)] expanded in guava (Table S17). Expression profiling analysis revealed that most genes involved in L‐galactose and recycling pathways were more highly expressed in fruits compared to other tissues, except one and two members of the MDHAR and AO families, respectively (Figure 5; Table S18). Genes encoding downstream biosynthesis enzymes exhibited a positive correlation between expression level and ascorbic acid content in fruits (Figure 5). This result is similar to a previous report in a closely related species, *Myrciaria dubia* (Myrtaceae) (Castro *et al*., 2015a). In other pathways, however, only one member of GalUR (D‐galacturonate reductase) and two of MIOX (myo‐inositol oxygenase) had detectable expression (Figure S16a). Taken together, these results indicate that the L‐galactose pathway is the major route for ascorbic acid biosynthesis in guava. In particular, MDHAR, rather than DHAR (dehydroascorbate reductase), contributes to ascorbic acid regeneration in guava.

In a weighted gene coexpression network analysis (WGCNA), we found that three of the 20 coexpression modules (Figure 4b) were positively correlated with ascorbic acid content (Figure 4c: tan, 0.91; yellow, 0.81; and light yellow, 0.74). Within these modules, we found six genes involved in ascorbic acid biosynthesis and regeneration pathways (Figure S17a, b, c; Table S19), indicating that these genes play key roles in ascorbic acid accumulation. Expression of these genes showed positive correlation with the stages of fruit development. Five of the six key genes (except the one in the regeneration pathway) also showed high expression levels in the stem which, therefore, may also have the capacity for ascorbic acid biosynthesis (De Tullio and Arrigoni, 2003).

### Fruit softening related metabolism

Guava fruit loses its firmness during ripening (Ali *et al*., 2004). In comparison with woody‐fruited species (e.g. *L. scoparium* and *E. grandis*), ripening of fleshy fruits is often associated with starch degradation or cell wall metabolism (Cordenunsi‐Lysenko *et al*., 2019; Wang *et al*., 2018). Here, we investigated genes involved in these two pathways.

**Figure 6:**
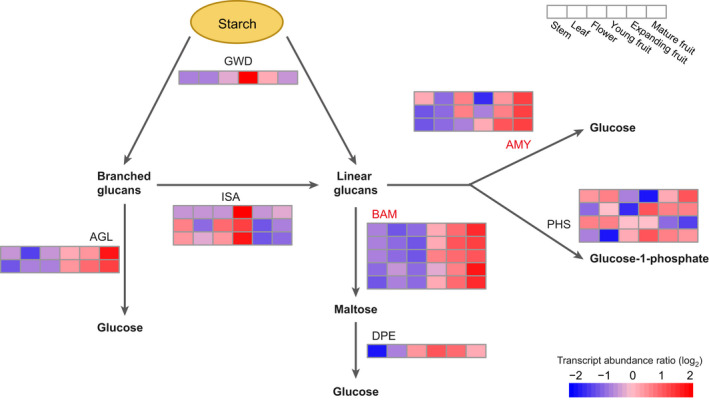
The proposed starch degradation pathways in plants and transcript abundance of their genes in guava. Gene families that expanded in guava are shown in red. The six boxes in one row of each heat map (left to right) correspond to the expression levels in stem, leaf, flower and at three fruit developmental stages. Each row in the heat map corresponds to one gene. AGL: α‐glucosidase; AMY: α‐amylase; BAM: β‐amylase; DPE: 4‐α‐glucanotransferase; GWD: glucan, water dikinase; ISA: isoamylase; PHS: glucan phosphorylase.

In the genome sequences of guava, we found that 44 members of seven gene families involved in the starch degradation pathway (Figure 6; Table S20). Compared with *E. grandis*, *P. granatum* and *A. thaliana* (Table S20), guava has the greatest number of genes in gene families related to starch degradation. The main degradation genes in guava are *AMY* (α‐amylase) and *BAM* (β‐amylase) genes, which account for 61.4% of these starch degradation genes in guava. We detected more members of *AMY* and *BAM* in guava than in any other plant that we surveyed. Within guava, we also detected α‐glucosidase (AGL), 4‐α‐glucanotransferase (DPE) and isoamylase (ISA), which are additional key enzymes in the starch degradation pathway, and these were not present in *E. grandis* or *P. granatum*.

We evaluated starch content in fruits at different developmental stages (Figure 4a; Table S16). Gene expression in 21 samples of *P. guajava* was clustered into 20 coexpression modules (Figure 4b), of which, two were significantly negatively correlated with starch content (Figure 4c: tan, −0.92; light yellow, −0.70). Within these two, we found four and one starch degradation genes, respectively (Figure S17a, b; Table S19). Most genes involved in starch degradation showed increased expression with fruit development in guava (Figure 6; Figure S18), indicating that starch degradation plays an important role in fruit softening during the ripening process.

We also identified a total of 192 genes encoding five key enzymes related to cell wall degradation (Table S21). Guava possesses more gene members of *β‐galactosidase* than other plants. We found many genes related to cell wall degradation with high expression levels in fruits, even though they did not show consistent trends during ripening (Figure S19). Some genes encoding β‐galactosidase, polygalacturonase and pectate lyase had increasingly higher expression during fruit ripening (Figure S19). This is in accord with prior work showing that the content of soluble pectin (i.e. galacturonic acid) increased with a reduction of guava fruits firmness (Braga *et al*., 2017). Together, these results indicate that cell wall degradation may also contribute to fruit softening in guava.

### Positive selection in guava, *L. scoparium* and *E. grandis*

In order to investigate the evolutionary footprint of natural selection on candidate genes associated with the ascorbic acid pathway and fruit softening, we conducted positive selection analyses for guava, *L. scoparium* and *E. grandis* using orthologs from *P. granatum* as the outgroup. Among the 15 681 orthogroups, 3627 contained single‐copy orthologous genes. In guava, we identified 285 positively selected genes (PSGs) (*P* < 0.01; Table S22). A GO functional classification of significant PSGs in guava revealed several categories associated with proteolysis (Figures S20 and S21). Among the 285 genes, we found two (Pgu12819 and Pgu22300) encoding *AMY* and two genes involved in starch biosynthesis (Pgu05347 and Pgu03782). Furthermore, three of these positively selected genes (Pgu03782, Pgu12819 and Pgu22300) were expressed increasingly during fruit ripening (Figures S16b and S18).

There were 859 and 313 genes possibly under positive selection in *L. scoparium* and *E. grandis* (*P* < 0.01), respectively. We found that four and three genes, which were involved in lignin biosynthesis pathways, were PSGs of *L. scoparium* and *E. grandis*, respectively (Table S23). These results are consistent with the hypotheses that woody‐fruit formation occurs via secondary cell wall synthesis and lignification (Dardick and Callahan, 2014). Together with the analyses of gene expression during fruit softening, these results support that fleshy‐fruited species in Myrtaceae underwent positive selection on starch metabolic genes, and the starch degradation may be the main contributor to fruit softening.

## Discussion

In terms of number of species, Myrtaceae is the 8th largest family of flowering plants, with 5950 species in 132 genera, which are distributed primarily across subtropical and tropical regions of the world (Christenhusz and Byng, 2016). The family includes many plants of economic value, such as species of *E. grandis*, *Eugenia uniflora* and *P. guajava*, which are important sources of timbers, essential oils, and fruits (Grattapaglia *et al*., 2012). Genomic resources can facilitate molecular breeding in guava and evolutionary studies of Myrtaceae. Here, we obtained a chromosome‐level, high‐quality genome assembly of guava (*P. guajava*) using PacBio in association with NGS sequencing and Hi‐C mapping. This nearly complete genome will be valuable for revealing the mechanisms of biosynthesis of economically valuable natural products of the species. The genome assembly will also be a useful genetic resource for molecular breeding and can contribute to improving strategies for postharvest storage.

The fruits of guava have among the highest natural concentrations of ascorbic acid (vitamin C), folic acid (vitamin B_9_), niacin (vitamin B_3_), pyridoxine (vitamin B_6_) and dietary fibre (Rishika and Sharma, 2012). An investigation of antioxidant activity in 19 popular tropical fruits has shown that guava had the highest ascorbic acid content (Guevara *et al*., 2019). In the present study, we predicted the ascorbic acid biosynthesis and recycling pathways in guava and identified candidate genes encoding enzymes in these pathways. Analyses of gene families demonstrated that the L‐galactose pathway may serve as a major route of ascorbic acid biosynthesis in guava. This mechanism is different from *M. dubia*, a closely related species of the same family, which possesses all four documented metabolic pathways for ascorbic acid biosynthesis (Castro *et al*., 2015b). This may indicate that the mechanisms of ascorbic acid biosynthesis are lineage‐specific in Myrtaceae. However, the L‐galactose pathway is the primary pathway for ascorbic acid biosynthesis in many other plants such as *Ziziphus jujube* (Rhamnaceae) and *Myrica rubra* (Myricaceae) (Feng *et al*., 2012; Liu *et al*., 2014). Interestingly, fruits of species that primarily use the L‐galactose pathway for ascorbic acid biosynthesis are all rich in ascorbic acid. This suggests that, in plants, having multiple biosynthesis pathways is neither required for nor predictive of producing high concentrations of ascorbic acid.

Ripening processes are different among fruit types and species (Li *et al*., 2019; Wang *et al*., 2018). Among fruits that exhibit the cell wall disassembly mechanism of fruit softening, pectin and pectin degrading enzymes have attracted the most attention as key cell wall components undergoing changes during maturation (reviewed in Uluisik and Seymour, 2020). For guava, prior studies were incongruent in their findings for pectin content in fruits and enzyme activities that degrade pectin. For example, Ali *et al*. (2004) reported that ripening of guava fruits accompanied by limited pectin degradation, but Abreu *et al*. (2012) and Braga *et al*. (2017) found that pectin plays an important role in the firmness of guava fruits. Our transcriptomic analysis represents the first investigation of gene expression profiles underlying pathways involved pectin degradation in different tissues and fruit developmental stages in guava. The RNA‐Seq analysis in this study supports that enzyme activity and pectin content change during fruit ripening in guava as reported by Braga *et al*. (2017). Recently, Chen *et al*. (2017) reported that postharvest softening in *S. samarangense*, another fleshy‐fruited species of Myrtaceae, was ascribed to cell wall degradation. Taken these results together, it seems that cell wall degradation contributes to fruit softening commonly in Myrtaceae. Similar mechanisms were also found in most fruits such as mango (Anacardiaceae), strawberry (Rosaceae) and pear (Rosaceae) (reviewed by Wang *et al*., 2018).

In addition to pectin, starch degradation is also considered a major pathway for fruit softening (Brady, 1987; Giovannoni *et al*., 1992). Expression patterns of genes involved in starch degrading in guava (Figure 6), overwhelmingly showed that starch content decreased with fruit ripening (Figure 4; Table S19). Collectively, these results may indicate that guava fruit softening is due to the joint action of the cell wall degradation and starch degradation. During fruit ripening, energy for producing volatile compounds is generally provided by glucose which is the main product of starch degradation (Cordenunsi‐Lysenko *et al*., 2019). The abundance of volatile metabolites in mature and soft guava fruit (Moon *et al*., 2018) may depend on the hydrolysis of starch, which protects cell membranes and maintains firmness. Starch degradation was also reported as playing a key role in fruit softening in banana (Musaceae) (Shiga *et al*., 2011; Song *et al*., 2019) and persimmon (Ebenaceae) (Jung *et al*., 2017). The reference genome of guava reported here will facilitate studies on both ripening and softening mechanisms and support efforts to prolong the shelf life of guava fruits.

Woody fruits, as the name implies, are highly lignified. As Carocha *et al*. (2015) reported, each enzyme in lignification pathways has coding genes expressed relatively highly in fruit capsules compared to other tissues. In our PSG analysis, genes in lignin biosynthesis pathways have undergone positive selection in woody‐fruited species of Myrtaceae (*L. scoparium* and *E. grandis*). In contrast, genes involving starch accumulation and degradation were found to be positively selected in guava. These results further support that woody fruits in Myrtaceae undergo lignin accumulation during maturation and fleshy fruits may be the result of starch biosynthesis and degradation. However, fleshy fruits in Myrtaceae have arisen more than once, and thus, more genomic or transcriptomic data for additional species of Myrtaceae are needed to gain a more comprehensive understanding of the evolution of fruit types in the family.

## Experimental procedures

### Sample preparation, de novo sequencing and assembly

For genome sequencing, we chose a mature healthy tree of the guava cultivar ‘New Age’ from the garden of Guangdong Ocean University (21.1526 N, 110.2975 E), Zhanjiang in Guangdong Province, China. We extracted genomic DNA from fresh leaves, using a modified CTAB–chloroform protocol (Doyle and Doyle, 1987) and constructed short‐insert libraries with a size of 500 bp for sequencing on the Illumina HiSeq 2500 platform under the PE 150 bp protocol. For PacBio library construction, we sheared the genomic DNA of *P. guajava* to 20 kb fragments, which we used to prepare a SMRT library for sequencing with the PacBio Sequel system (Chin *et al*., 2013). We used fresh young leaves of *P. guajava* to construct Hi‐C sequencing library. In brief, cross‐linked chromatin was digested with *Dpn* II and ligated *in situ* after biotinylation. DNA fragments were enriched via the interaction of biotin and blunt‐end ligation and then subjected to Illumina NovaSeq PE150 sequencing (Belton *et al*., 2012).

We applied the FALCON v0.3.0 (Chin *et al*., 2016) to correct errors in PacBio long reads according to PacBio short reads (<5 kb) and then generated consensus sequences. We assembled the primary scaffolds with these subreads, and we further corrected the scaffolds in PILON v1.22 (Walker *et al*., 2014) using the short sequencing reads. To anchor scaffolds onto chromosomes, we aligned the Hi‐C sequencing data to the assembly by BWA v0.7.17 (Li and Durbin, 2009) and detected valid contacts. Preassembled scaffolds were clustered, ordered and directed onto pseudochromosomes with LACHESIS software (Hariharan and Toyama, 2004). To increase the accuracy of the assembled genome, we artificially corrected the LACHESIS‐based assembly, filled gaps and removed duplicate sequences (Burton *et al*., 2013).

### Genome quality assessment

We estimated the genome size based on k‐mer distribution analysis with the programme in GCE (Liu et al., 2013) using Illumina short reads. We used BUSCO (Simão *et al*., 2015) and CEGMA (Parra *et al*., 2007) to evaluate the completeness and accuracy of the genome assembly and BWA v0.7.17 (Li, 2014) to align the Illumina short reads to the assembly and evaluate the assembled portion. We assembled expressed sequencing tags (ESTs) using Trinity v2.8.4 (Grabherr *et al*., 2011) with RNA reads from five different tissues (stems, leaves, flowers, fruits and seeds). To further examine the quality of the genome assembly, we aligned ESTs to the assembled genome using BLAT (Kent, 2002) using parameters of identity ≥90% and coverage ≥50%. We also evaluated the quality of the genome assembly by mapping RNA‐Seq reads from the five different tissues to the ESTs using TopHat2 (Trapnell *et al*., 2012).

### Genome annotations

We identified repetitive sequences in *P. guajava* at both the DNA and protein levels by integrating homology‐based prediction and *de novo* identification. We performed repeat masking based on repeats in the Repbase TE library from the repbase server (Bao *et al*., 2015). Using this library, we predicted interspersed repeat elements through RepeatMasker v3.3.0 and ProteinMask (Smit *et al*., 1996‐2010) and screened tandem repeats through TRF v4.07b (Benson, 1999).

We conducted annotation of protein‐coding genes in the *P. guajava* genome using a combination of *de novo* gene prediction, homology‐based prediction and RNA‐seq‐based prediction. For *de novo* identification, we predicted the gene models by five *ab initio* gene prediction programmes, Augustus (Stanke *et al*., 2006), GlimmerHMM (Majoros *et al*., 2004), SNAP (Korf, 2004), Genscan (Burge and Karlin, 1997) and Geneid (Guigó *et al*., 1992), respectively. We aligned proteins from five sequenced plants, that is *A. thaliana* (Kaul *et al*., 2000), *Citrus sinensis* (Wang *et al*., 2017), *E. grandis* (Myburg *et al*., 2014), *P. granatum* (Qin *et al*., 2017), and *Theobroma cacao* (Argout *et al*., 2011), to the *P. guajava* assembly using tBlastN (Altschul *et al*., 1990) with an e‐value cut‐off of 1 × 10^−5^. Gene models were generated by Genewise v2.2.0 (Birney *et al*., 2004). We applied two methods of RNA‐Seq‐based prediction: (i) We mapped RNA‐Seq data to the genome using TopHat2 (Kim *et al*., 2013) and further generated the gene models in Cufflinks (Trapnell *et al*., 2012) based on exons, and (ii) We aligned ESTs against the assembly to generate the gene models using PASA (Haas *et al*., 2003). We integrated predictions from the three approaches in EVidenceModeler v1.1.1 (Haas *et al*., 2008) to generate a non‐redundant gene model set. We annotated the final gene model using the non‐redundant protein database of NCBI (NR), Swissprot, KEGG (Kanehisa *et al*., 2016), Interprot (Quevillon *et al*., 2005) and Pfam databases.

### Phylogenetic analysis and estimation of divergence time

We used OrthoFinder v2.2.7 (Emms and Kelly, 2015) to identify orthologous genes from guava and ten other species of angiosperms including Arabidopsis (*A. thaliana*), eucalyptus (*E. grandis*), mānuka (*L. scoparium*), apple (*Malus domestica*), peach (*Prunus persica*), papaya (*Carica papaya*), pomegranate (*P. granatum*), tomato (*S. lycopersicum*), common wine grape (*V. vinifera*) and rice (*Oryza sativa*). We performed alignment of proteins of single‐copy orthologous genes with MUSCLE v3.8.425 (Edgar, 2004). Based on these alignments, we used IQ‐TREE v1.6.11 (Nguyen *et al*., 2015) to estimate a maximum‐likelihood (ML) phylogenetic tree for each gene and ASTRAL‐II v5.6.3 (Mirarab and Warnow, 2015) to estimate the species tree by summarizing across gene trees. We used Bayesian Evolutionary Analysis Sampling Trees (BEAST) v2.6.0 (Drummond *et al*., 2012) to estimate species divergence times based on a split between Arabidopsis and papaya (mean: 71.9 MYA. Std dev: 2 MYA) (Wikström *et al*., 2001) as a secondary calibration. We ran the Markov chain Monte Carlo for 100 000 000 generations with sampling every 1000 generations.

### Gene family expansion and contraction analysis

To study gene family expansion and contraction, we undertook an analysis of gene family sizes using CAFÉ v4.2 (De Bie *et al*., 2006). We obtained counts of gene families and genes from OrthoFinder. We determined the gene family expansions or contractions only when the change in gene copy number was significant with *P* < 0.01.

### Whole‐genome alignment and WGD analysis

To assess the degree of collinearity, we used the python version of MCScan (Tang *et al*., 2008) to identify syntenic blocks between guava, *E. grandis* and *V. vinifera*. We defined a syntenic region as one containing a minimum of 30 shared genes. In addition, we constructed a circos map for guava, *L. scoparium* and *E. grandis*.

For analysis of the WGD events, we applied the python library, ‘wgd’, (Zwaenepoel and Van de Peer, 2019) to construct *K*s‐based age distributions. In brief, we performed one‐to‐one comparisons of orthologs using BLASTP with an e‐value cut‐off of 1 × 10^−10^. We obtained *K*s values for all gene pairs through ML estimation in CODEML (https://svn.omdoc.org/repos/codeml/doc/spec/codeml) of the PAML package (Yang, 2007). To visualize the *K*s distributions, we used kernel density estimates, and we fitted *K*s distribution curves with Gaussian mixture models.

To infer and locate putative WGD events in Myrtales, we used the Multi‐tAxon Paleopolyploidy Search (MAPS) tool (Li *et al*., 2015). We selected seven species, including four species of Myrtales (*P. guajava*, *L. scoparium*, *E. grandis*, *P. granatum*) that potentially share a WGD in their ancestry and three additional representative species of eudicots (*A. thaliana*, *V. vinifera* and *S. lycopersicum*). MAPS uses a user‐defined species tree, which resulted from our phylogenetic analysis, to filter collections of gene trees for subtrees consistent with relationships at each node in the species tree. Gene trees were also constructed with IQ‐TREE v1.6.11 (Nguyen *et al*., 2015).

The ages of WGDs detected were estimated under the assumption that synonymous mutations are accumulated at a constant rate. According to the formula divergence data = *K*s/(2 × *r*) (Badouin *et al*., 2017; Vanneste *et al*., 2013), we inferred *r* (plant average *K*s/year rate) in Myrtales via *K*s distributions of paralogous genes. We then applied the average *K*s/year rate to estimate WGD ages of each sampled species of Myrtales.

### Ascorbic acid and starch content determinations

For the investigation of fruit development, we collected samples of young fruits (two weeks after fruit set), expanding fruits (five weeks after fruit set) and mature fruits (eight weeks after fruit set). For each fruit stage, we made six independent collections from the individual tree we sequenced the genome.

The content of ascorbic acid was measured using the dinitrophenylhydrazine method (Otles, 1995). Briefly, frozen grounded samples were homogenized in metaphosphoric acid. The homogenate was centrifuged, and then, the supernatant was filtered through a Millipore membrane to measure total ascorbate. The ascorbate extracts were analysed by HPLC (high‐performance liquid chromatography) using an SB‐aq column (Agilent) eluted with acetate buffer (0.2 mol/L pH 4.5) at a flow rate of 1.0 mL/min. Elutes were detected at 254 nm, and a standard curve from 2 to 40 μg/mL ascorbic acid was obtained.

We extracted total starch from frozen, ground samples for each replicate following using a total starch assay kit (Megazyme International Ireland Ltd., Wicklow, Ireland), as described by Peris‐Tortajada (2018). In brief, we removed fats from frozen grounded samples with n‐hexane, followed by a further extraction with 80% aqueous methanol to remove soluble sugars. The resulting residue was gelatinized in boiling water and then incubated with pH 4.8 acetate buffer and amyloglucosidase. In this way, the starch was converted into glucose, which, after centrifugation, remains in the supernatant. We measured the glucose content with HPLC using a 5 μm Spherisorb NH_2_ column, a RI detector, and 85:15 v/v acetonitrile/water as mobile phase. We determined starch concentration by Starch (%) = 0.90 × 100 × C (mg/mL)/W (mg), where C is the content of glucose determined by HPLC and W is the mass of sample.

### Identification of gene families in related pathways

We downloaded protein sequences of gene families in ascorbic acid, starch and cell wall metabolism pathways in *A. thaliana* from the TAIR database and used these as queries in BLASTP searches against the guava protein sequences to identify homologous sequences. We checked KOG and KEGG annotations of these homologous genes and retained only those genes with hits having KOG and KEGG annotations.

### RNA sequencing and WGCNA analysis

From each collected fruit representing fruit developmental stages, we isolated and purified total RNA using TRIzol reagent (OMEGA Bio‐Tek, Shanghai, China) following the manufacturer’s instructions. Based on the purified total RNA, we generated RNA‐Seq libraries and sequenced these using an Illumina HiSeq 2000 system. We trimmed the resulting paired‐end reads to remove adaptors and enhance quality, and we removed trimmed reads that were <100 bp in size. We mapped the remaining reads to the *de novo* assembled genome of guava using TopHat2 (Trapnell *et al*., 2012) under default parameters. We assembled the mapped reads for each sample with Cufflinks (Trapnell *et al*., 2012) and then merged the assembled contigs with the reference gene annotations into a unified annotation, which we used to quantify gene expression in each sample. We normalized gene expression levels according to FPKM (fragments per kilobase exon model per million mapped fragments) and constructed coexpression networks using WGCNA v1.66 (Langfelder and Horvath, 2008). We calculated WGCNA module eigengene values and correlations between them and ascorbic acid and starch contents at different fruit developmental stages. We identified the functions of key genes in each inferred module by both annotation information and manual blast against genes of *A. thaliana* and *E. grandis*. Gene networks were visualized with Cytoscape v3.7.2 (Shannon *et al*., 2003).

### Positive selection analysis

For positive selection analysis, we first identified single‐copy orthologous genes from guava and the three most closely related species with assembled genomes: *L. scorparium* (Myrtaceae), *E. grandis* (Myrtaceae) and *P. granatum* (pomegranate, Lythraceae). For these genes, based on the phylogenetic topology, we employed the branch‐site model incorporated in the PAML package v4.9 (Zhang *et al*., 2005) to detect positively selected genes (PSGs). When one of the three species of Myrtaceae was specified as a foreground branch, the other two and the pomegranate branches in the phylogenetic tree were used as background branches. We conducted likelihood ratio tests to determine whether positive selection was operating on the foreground branch. In this study, PSGs were identified only when *P* < 0.01.

## Conflict of interest statement

The authors declared that they have no conflict of interest to this work.

## Author contributions

MK and YL conceived the project. MK, YL and Chen Feng designed the study. Chen Feng and Chao Feng performed the sampling and experiments, and data analysis. Chen Feng designed and visualized the figures. Chen Feng and MK wrote the manuscript. All authors read and approved the final manuscript.

## Supporting information


**Figure S1** K‐mer frequency distribution curve (*k* = 17) of Illumina short reads of the guava genome.
**Figure S2** Hi‐C contact data mapped to the genome of guava. The heat map represents the normalized contact matrix. The strongest and weakest contacts are shown in red and yellow, respectively.
**Figure S3** Prediction and annotation of genes in guava genome. (a) Number of genes predicted with *de novo*, homolog and RNA‐seq. All predicted genes were integrated by EVM. (b) Number of genes annotated with databases of Swissprot, NR, GO, KEGG, Pfam and InterPro.
**Figure S4** Diagram showing the gene ontology (GO) categories of the annotated genes in the guava genome.
**Figure S5** (a) The distribution of miRNA, rRNA, snRNA and tRNA genes on the guava pseudochromosomes. (b) The heat map of SSR distribution on the guava pseudochromosomes.
**Figure S6** Venn diagram showing orthologous groups shared among guava (*P. guajava*), *L. scoparium*, *E. grandis*, *P. granatum* and other species. Each number represents the number of gene families.
**Figure S7** Gene ontology enrichment of genes from expanded gene families in guava. Directed acyclic graph showed top enriched GO terms belonging to Category Biological Process. Rectangles indicate the significant terms with *P*‐value < 0.01, with colour ranging from dark red (represent most significant *P*‐value) to bright yellow (least significant). The information displayed for each node, from first line to fourth line, is the GO term, GO name, *P*‐value and the number of duplicates from the D event/ the number of total genes annotated to the respective GO term, respectively.
**Figure S8** Gene ontology enrichment of genes from expanded gene families in guava. Directed acyclic graph showed top enriched GO terms belonging to Category Molecular Function.
**Figure S9** Gene ontology enrichment of genes from contracted gene families in guava. Directed acyclic graph showed top enriched GO terms belonging to Category Biological Process.
**Figure S10** Gene ontology enrichment of genes from contracted gene families in guava. Directed acyclic graph showed top enriched GO terms belonging to Category Molecular Function.
**Figure S11** Maximum‐likelihood species trees obtained using protein and DNA sequences of 3454 single‐copy orthologs. Support values are shown adjacent to nodes.
**Figure S12** *K*s distribution for paralogs in guava, *L. scoparium*, *E. grandis* and *P. granatum* (a), and for orthologs between them (b). Dashed lines in (a) which represent individual WGDs are fitted by a mixture model (BGMM).
**Figure S13** Syntenic blocks shared between the guava and *L. scoparium* genomes, and between guava and *E. grandis* genomes.
**Figure S14** MAPS result for potential WGDs. Percentage of subtrees indicates percentage of duplicates shared by descendant species at each node. The yellow star represents the WGD event shared by species of Myrtaceae.
**Figure S15** The four proposed ascorbic acid biosynthesis pathways in higher plants. Gene abbreviations are shown in Table S17.
**Figure S16** Heat map of gene transcript abundance in the ascorbic acid biosynthesis pathways (a) and starch biosynthesis (b) in different tissues and at different fruit developmental stages in *P. guajava*. FPKM values are log_2_‐based. Red and blue indicate high and low expression levels, respectively. GalUR: D‐galacturonate reductase; MIOX: myo‐inositol oxygenase; SS: starch synthase.
**Figure S17** Gene networks of the tan (a), light yellow (b) and yellow (c) modules. Candidate genes in starch degradation and ascorbic acid biosynthesis pathways are shown in green and red coloured circles, respectively. Genes and their abbreviations are shown in Tables S17, S19 and S20.
**Figure S18** Heat map of gene transcript abundance in the starch degradation pathway in different tissues and at different fruit developmental stages in *P. guajava*. FPKM values are log_2_‐based. Red and blue indicate high and low expression levels, respectively. Gene abbreviations are shown in Table S20.
**Figure S19** Heat map of important genes transcript abundance in the cellulose degradation and cell wall softening pathways in different tissues and at different fruit developmental stages in guava. Gene abbreviations are shown in Table S21.
**Figure S20** Gene ontology enrichment of positively selected genes in guava. Directed acyclic graph showed top enriched GO terms belonging to the Biological Process category.
**Figure S21** Gene ontology enrichment of positively selected genes in guava. Directed acyclic graph showed top enriched GO terms belonging to the Molecular Function category.

**Table S1** Summary statistics for the final genome assembly of *P. guajava*.
**Table S2** Evaluation of the genome assembly of *P. guajava* using core Eukaryotic genes mapping approach (CEGMA).
**Table S3** Evaluation of the genome assembly of *P. guajava* using Benchmarking Universal Single‐Copy Orthologs (BUSCO).
**Table S4** Assessment of genome assembly of *P. guajava* using EST sequences.
**Table S5** Statistics of the guava RNA‐Seq data from different tissues and developmental stages.
**Table S6** Summary of transposable elements in *P. guajava*.
**Table S7** Summary statistics of predicted protein‐coding genes in *P. guajava*.
**Table S8** Summary of protein‐coding gene annotation of *P. guajava*.
**Table S9** Non‐coding RNAs predicted in the genome of *P. guajava*.
**Table S10** List of plant genome sequences used in the comparative genomic analysis.
**Table S11** Gene families clustered by OrthoFinder in 11 species. Genes used for OrthoFinder were proteins without splice variants.
**Table S12** Gene Ontology enrichment analysis of species‐specific single‐copy genes in guava.
**Table S13** Gene Ontology enrichment analysis of genes in significantly expanded gene families in *P. guajava*.
**Table S14** Gene Ontology enrichment analysis of genes in significantly contracted gene families in *P. guajava*.
**Table S15** Multi‐tAxon Paleopolyploidy Search (MAPS) results on the portion of the phylogeny surrounding potential WGDs. Percentage of subtrees indicates percentage of duplicates shared by descendant species at each node. Node numbers correspond to species tree in Figure S14.
**Table S16** Starch and ascorbic acid content at different developmental stages in fruits of guava.
**Table S17** Number of predicted genes encoding enzymes of ascorbic acid biosynthesis and regeneration in guava, *E. grandis*, *P. granatum* and *A. thaliana*.
**Table S18** Gene of FPKM ≥ 5 in ascorbic acid biosynthesis and regeneration pathways.
**Table S19** Genes associated with ascorbic acid biosynthesis and starch degradation located in WGCNA coexpression modules.
**Table S20** Number of predicted genes encoding enzymes of starch degradation in guava, *E. grandis*, *P. granatum* and *A. thaliana*.
**Table S21** Number of predicted genes encoding enzymes involving cellulose degradation and cell wall softening in guava, *E. grandis*, *P. granatum* and *A. thaliana*.
**Table S22** Positively selected genes in the genome of *P. guajava*. Two *AMY* genes and two genes involved in starch biosynthesis are in bold.
**Table S23** Positively selected genes in the lignin biosynthesis pathway in the genomes of *L. scoparium* and *E. grandis*.

## Data Availability

The raw genomic Illumina sequences, PacBio sequences and transcriptome data have been deposited in the NCBI Sequence Read Archive under accession numbers PRJNA631442. The phenotypic data are deposited in Figshare (10.6084/m9.figshare.12277934.v1).
