# Supplementary material for: A chromosome‐level genome assembly provides insights into ascorbic acid accumulation and fruit softening in guava (Psidium guajava)
Source: Plant Biotechnol J. 2020 Nov 12;19(4):717–30. doi: 10.1111/pbi.13498 (PMC8051600; doi:10.1111/pbi.13498)
Supplement: Supplementary file 1 — Figure S1 K‐mer frequency distribution curve (k = 17) of Illumina short reads of the guava genome. Figure S2 Hi‐C contact data mapped to the genome of guava. The heat map represents the normalized contact matrix. The strongest and weakest contacts are shown in red and yellow, respectively. Figure S3 Prediction and annotation of genes in guava genome. (a) Number of genes predicted with de novo, homolog and RNA‐seq. All predicted genes were integrated by EVM. (b) Number of genes annotated with databases of Swissprot, NR, GO, KEGG, Pfam and InterPro. Figure S4 Diagram showing the gene ontology (GO) categories of the annotated genes in the guava genome. Figure S5 (a) The distribution of miRNA, rRNA, snRNA and tRNA genes on the guava pseudochromosomes. (b) The heat map of SSR distribution on the guava pseudochromosomes. Figure S6 Venn diagram showing orthologous groups shared among guava (P. guajava), L. scoparium, E. grandis, P. granatum and other species. Each number represents the number of gene families. Figure S7 Gene ontology enrichment of genes from expanded gene families in guava. Directed acyclic graph showed top enriched GO terms belonging to Category Biological Process. Rectangles indicate the significant terms with P‐value < 0.01, with colour ranging from dark red (represent most significant P‐value) to bright yellow (least significant). The information displayed for each node, from first line to fourth line, is the GO term, GO name, P‐value and the number of duplicates from the D event/ the number of total genes annotated to the respective GO term, respectively. Figure S8 Gene ontology enrichment of genes from expanded gene families in guava. Directed acyclic graph showed top enriched GO terms belonging to Category Molecular Function. Figure S9 Gene ontology enrichment of genes from contracted gene families in guava. Directed acyclic graph showed top enriched GO terms belonging to Category Biological Process. Figure S10 Gene ontology enrichment of genes from [file PBI-19-717-s001.docx]

**
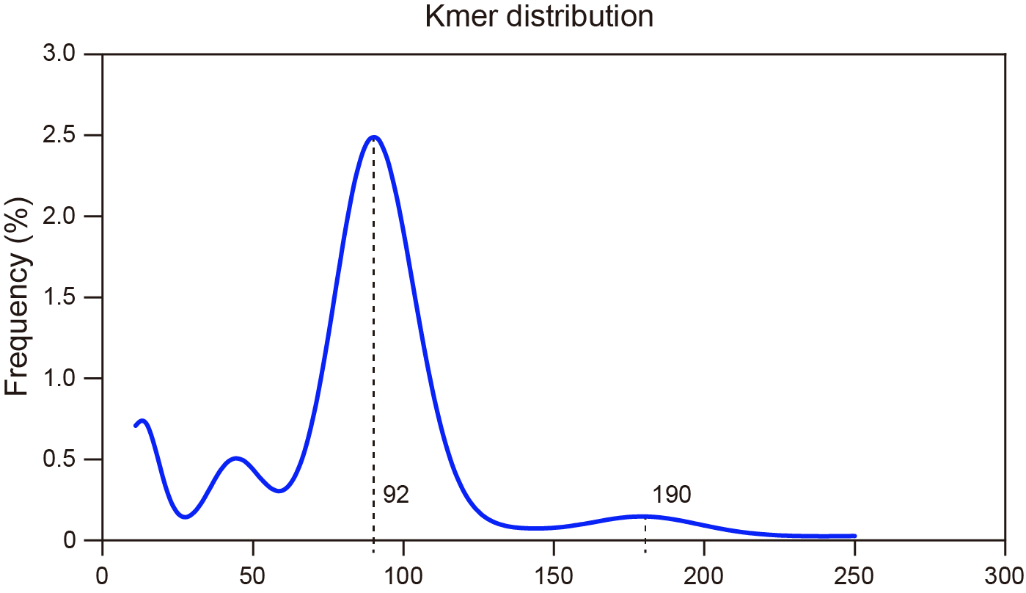
**

**Figure S1.** K-mer frequency distribution curve (k =17) of Illumina short reads of the guava genome.

**
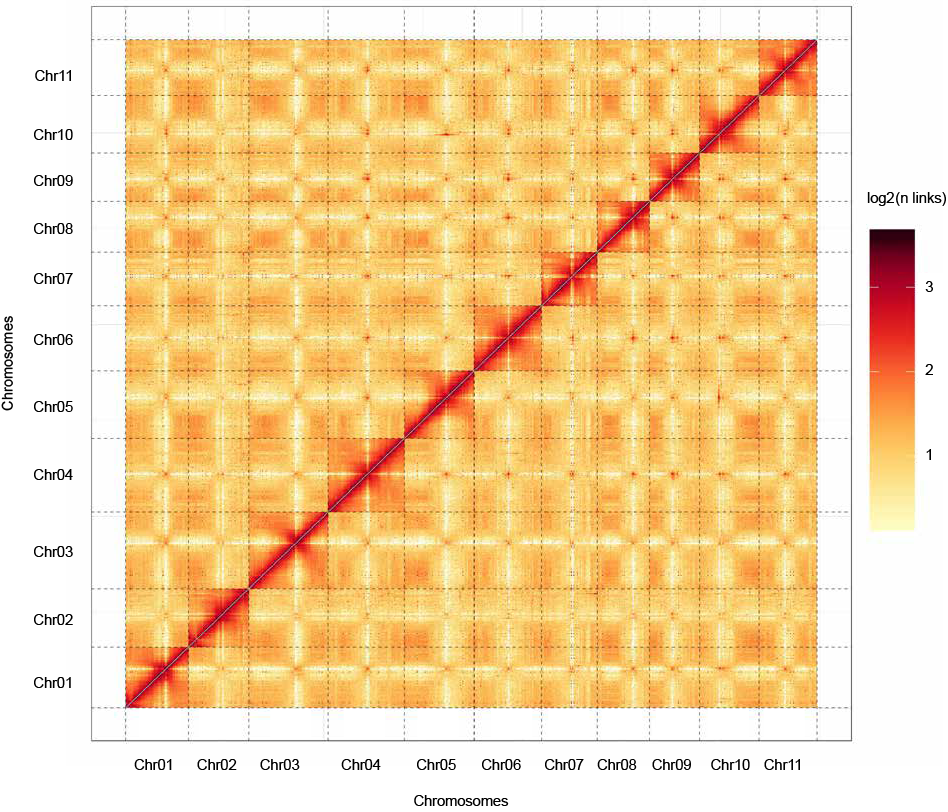
**

**Figure S2.** Hi-C contact data mapped to the genome of guava. The heat map represents the normalized contact matrix. The strongest and weakest contact are shown in red and yellow, respectively.

**
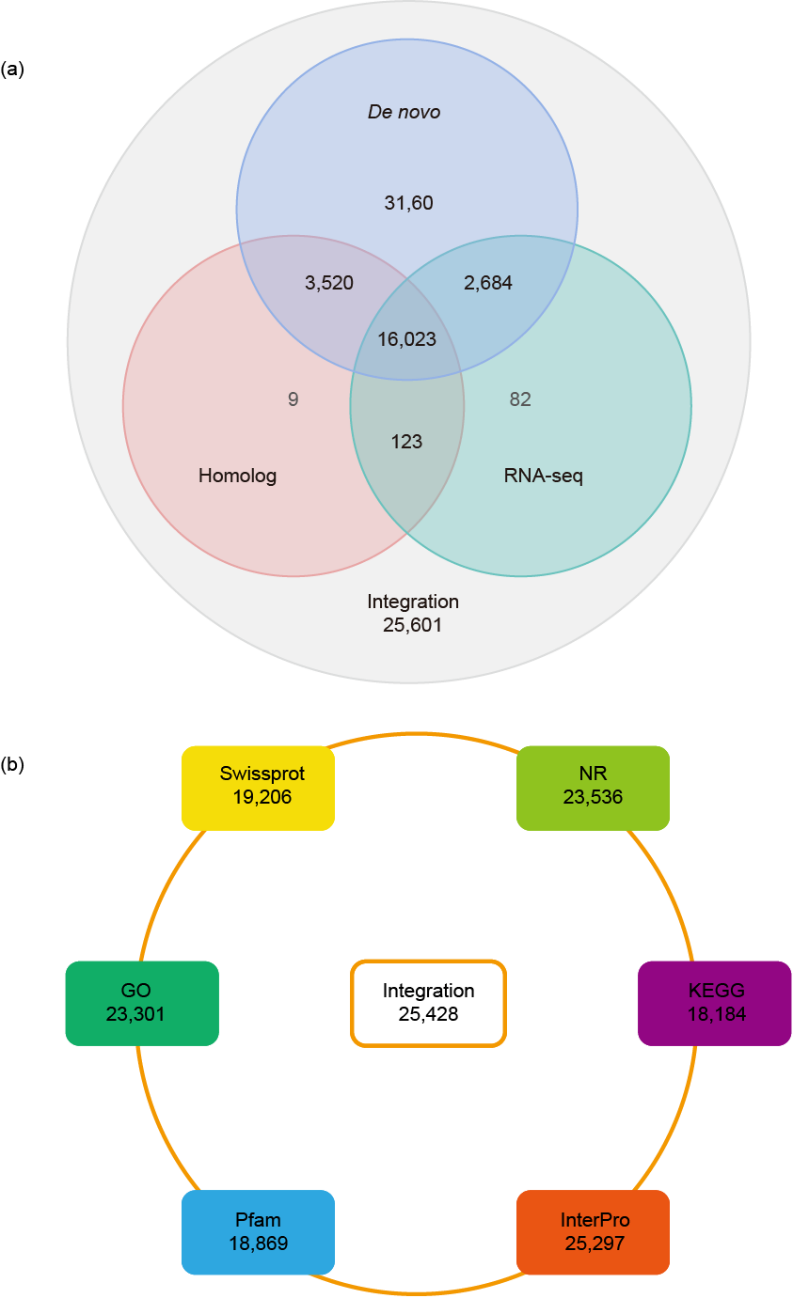
**

**Figure S3.** Prediction and annotation of genes in guava genome. (a) Number of genes predicted with *de novo*, homolog, and RNA-seq. All predicted genes were integrated by EVM. (b) Number of genes annotated with databases of Swissprot, NR, GO, KEGG, Pfam and InterPro.

**
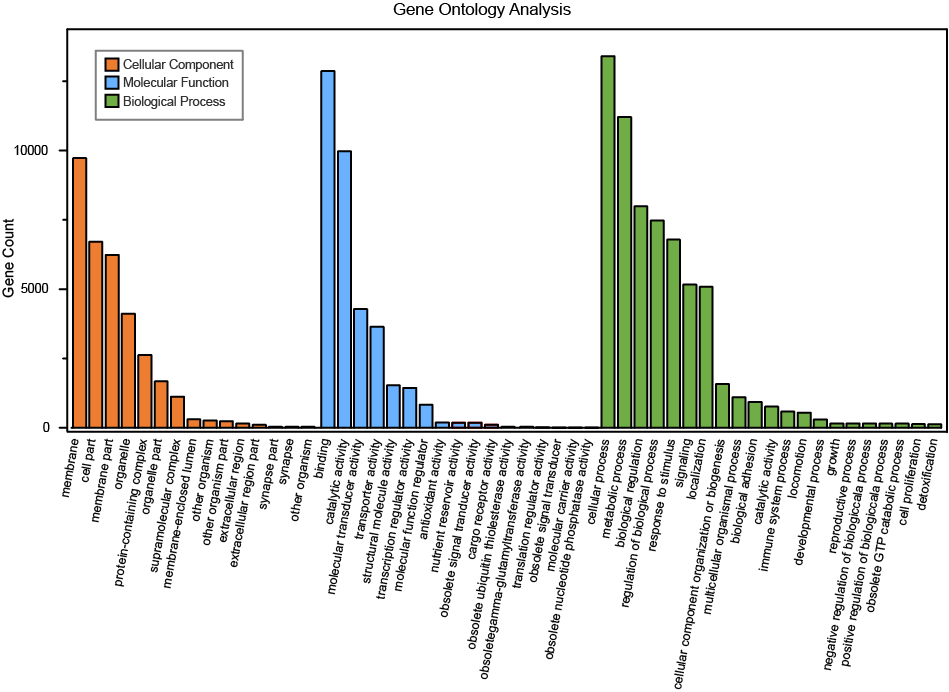
**

**Figure S4.** Diagram showing the gene ontology (GO) categories of the annotated genes in the guava genome.

**
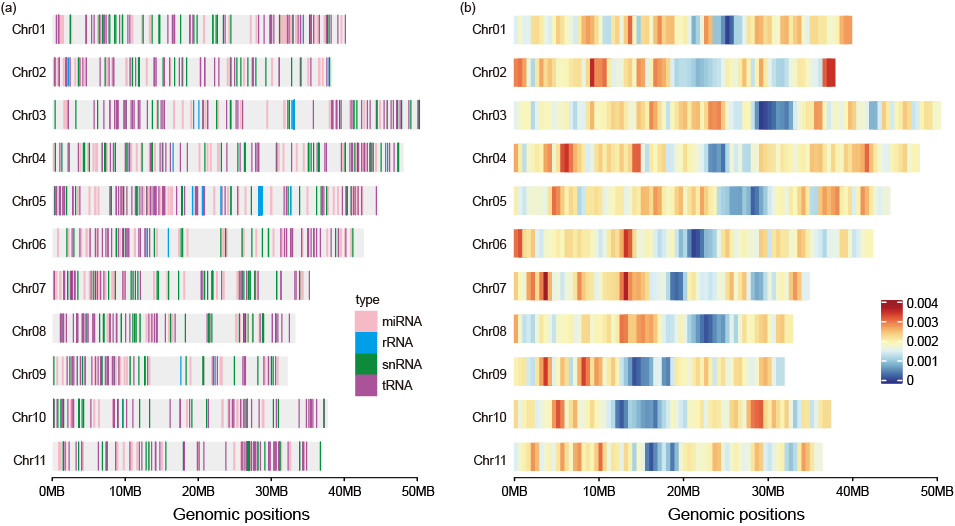
**

**Figure S5.** (a) The distribution of miRNA, rRNA, snRNA, and tRNA genes on the guava pseudochromosomes. (b) The heat map of SSR distribution on the guava pseudochromosomes.

**
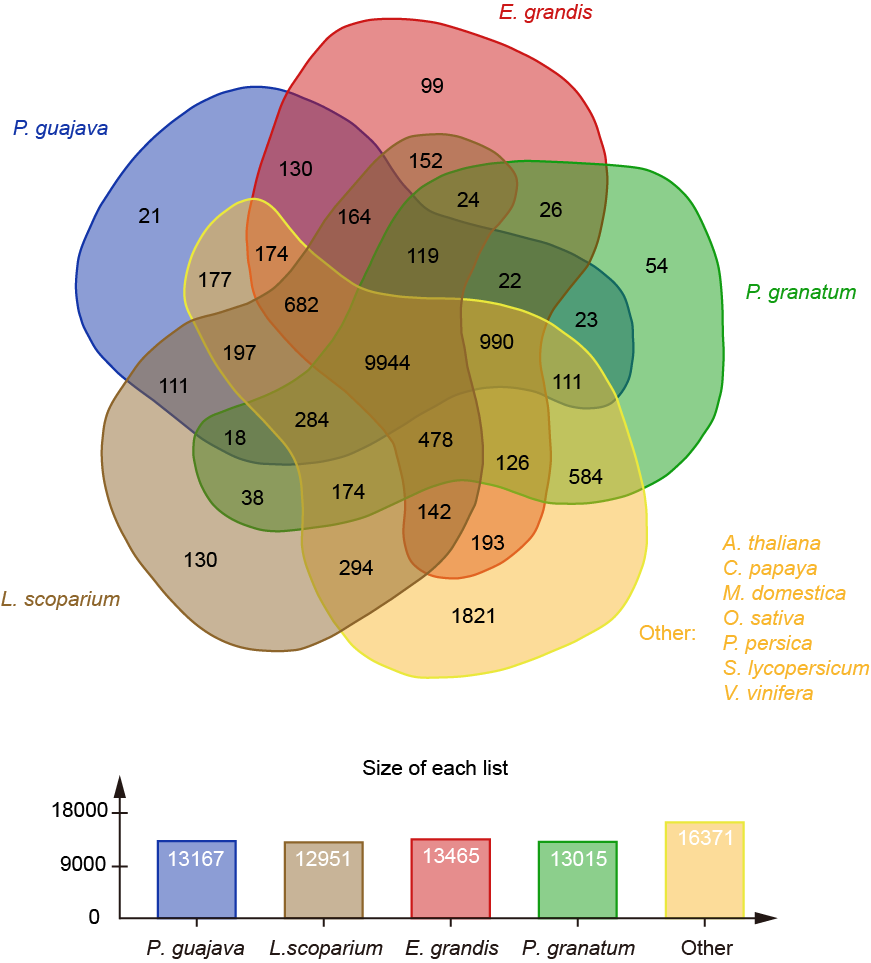
**

**Figure S6.** Venn diagram showing orthologous groups shared among guava (*P. guajava*), *L. scoparium*, *E. grandis*, *P. granatum*, and other species. Each number represents the number of gene families.

**
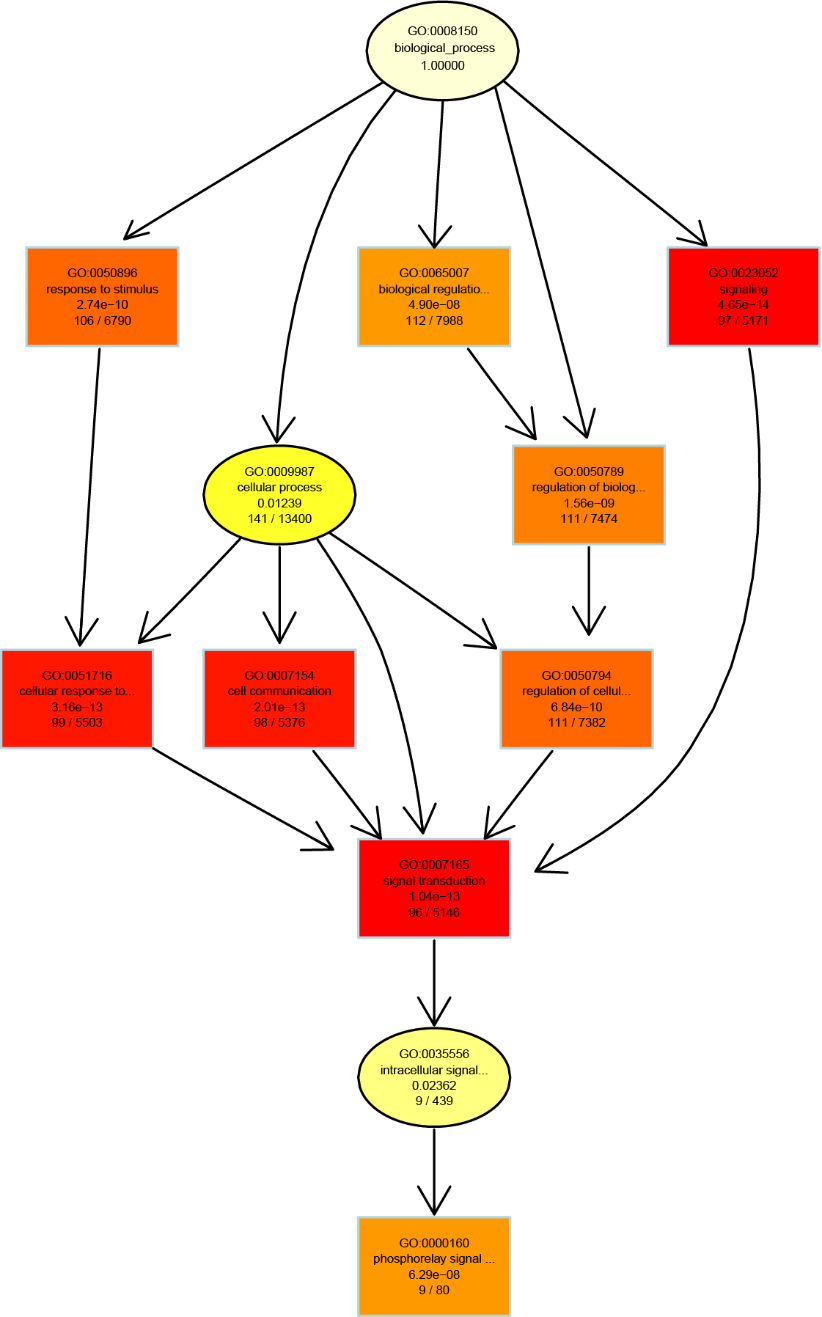
**

**Figure S7.** Gene ontology enrichment of genes from expanded gene families in guava. Directed acyclic graph showed top enriched GO terms belonging to Category Biological Process. Rectangles indicate the significant terms with *p*-value < 0.01, with color ranging from dark red (represent most significant *p*-value) to bright yellow (least significant). The information displayed for each node, from first line to fourth line, is the GO term, GO name, *p*-value, and the number of duplicates from the D event / the number of total genes annotated to the respective GO term, respectively.

**
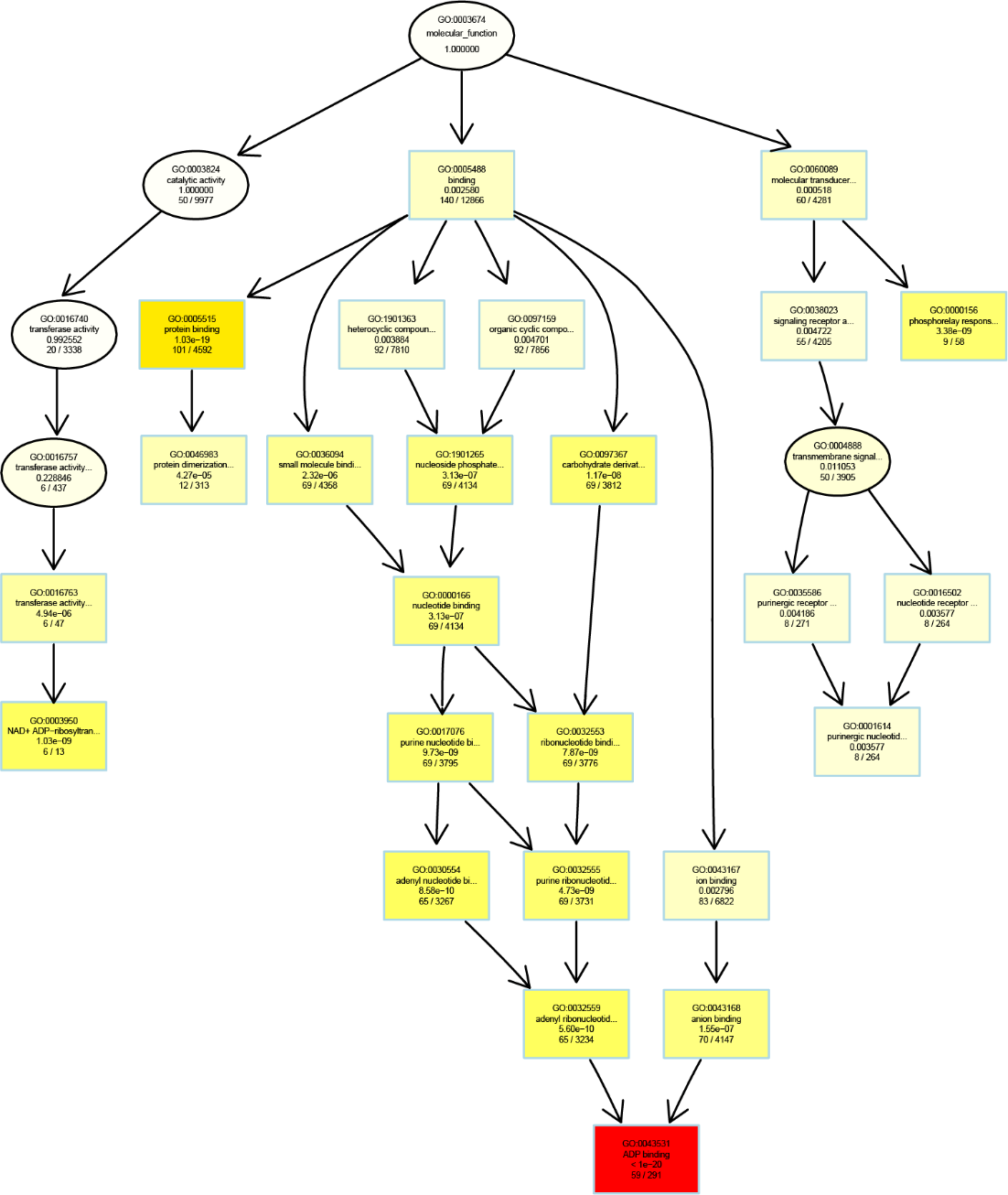
**

**Figure S8.** Gene ontology enrichment of genes from expanded gene families in guava. Directed acyclic graph showed top enriched GO terms belonging to Category Molecular Function.

**
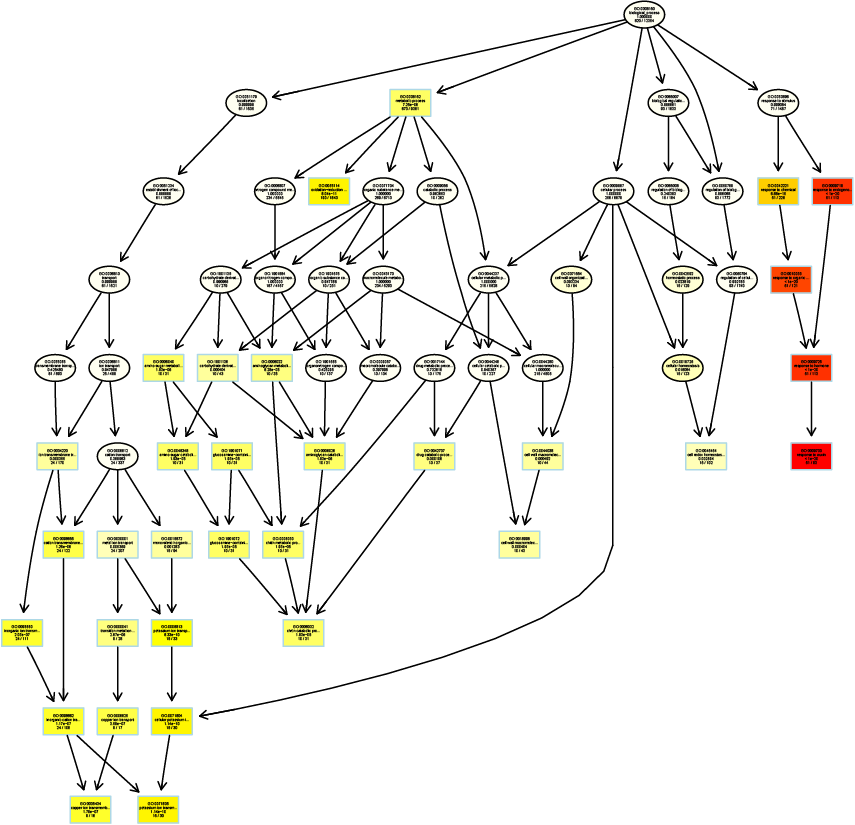
**

**Figure S9.** Gene ontology enrichment of genes from contracted gene families in guava. Directed acyclic graph showed top enriched GO terms belonging to Category Biological Process.

**
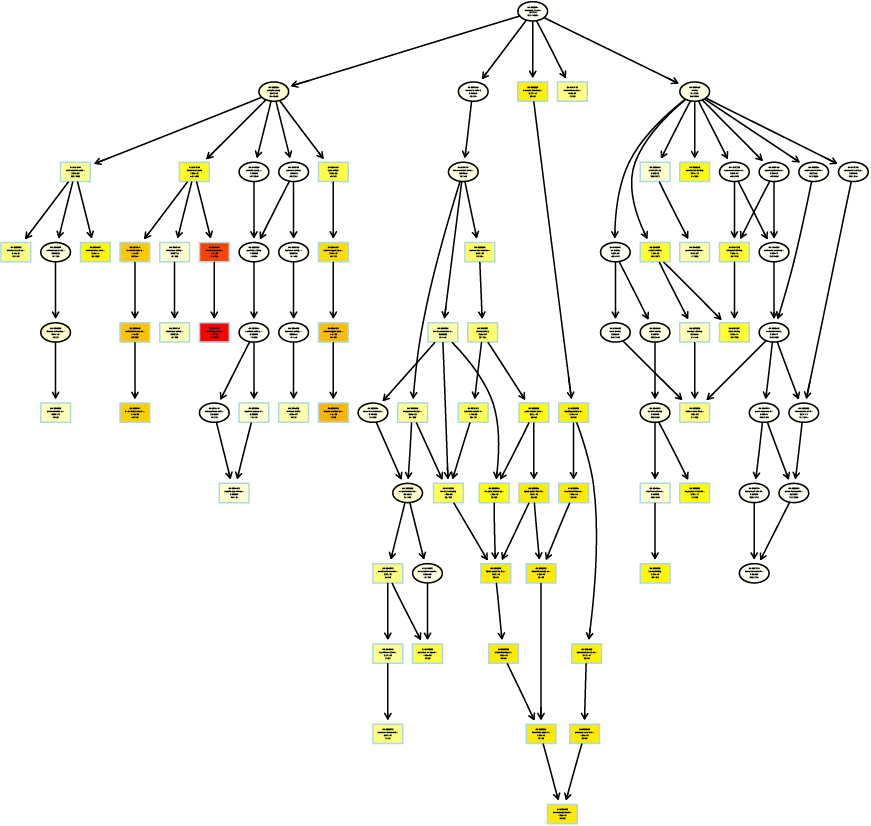
**

**Figure S10.** Gene ontology enrichment of genes from contracted gene families in guava. Directed acyclic graph showed top enriched GO terms belonging to Category Molecular Function.

**
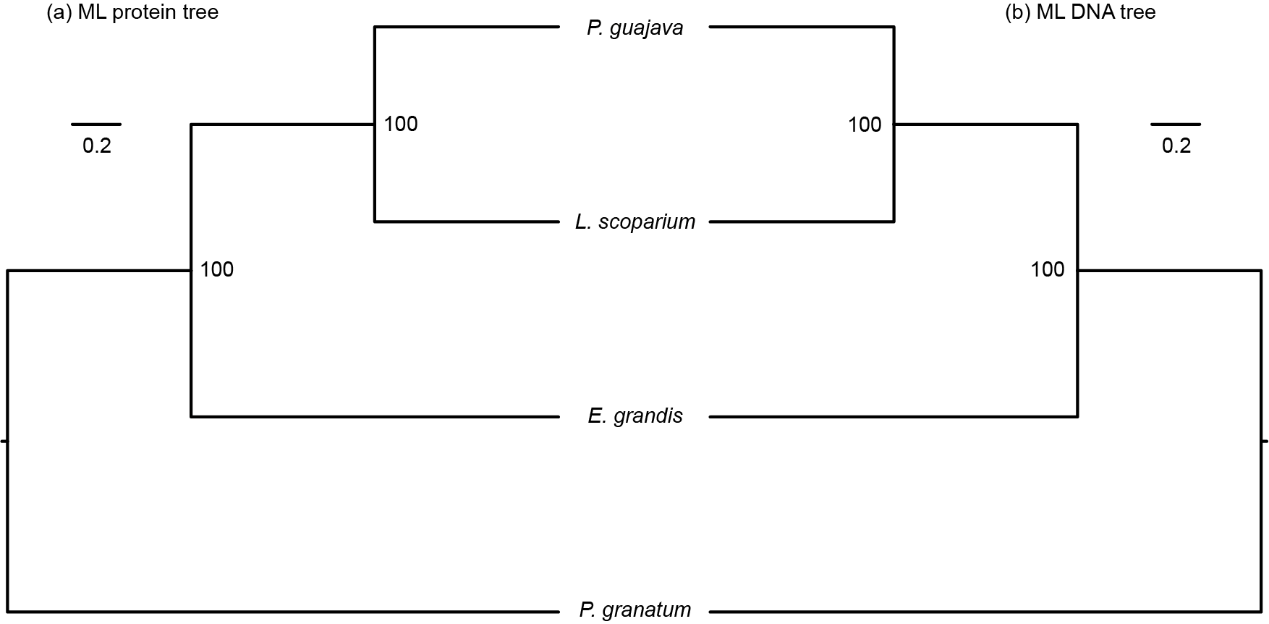
**

**Figure S11.** Maximum likelihood species trees obtained using protein and DNA sequences of 3454 single-copy orthologs. Support values are shown adjacent to nodes.

**
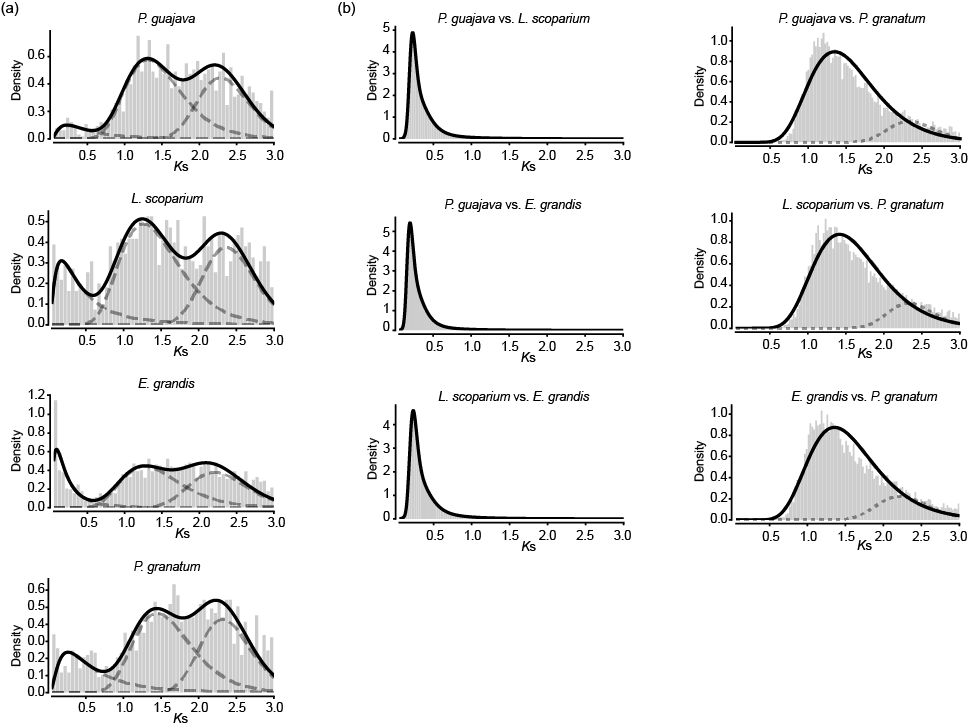
**

**Figure S12.** *K*s distribution for paralogs in guava, *L. scoparium*, *E. grandis*, and *P. granatum* (a), and for orthologs between them (b). Dashed lines in (a) which represent individual WGDs are fitted by a mixture model (BGMM).

**
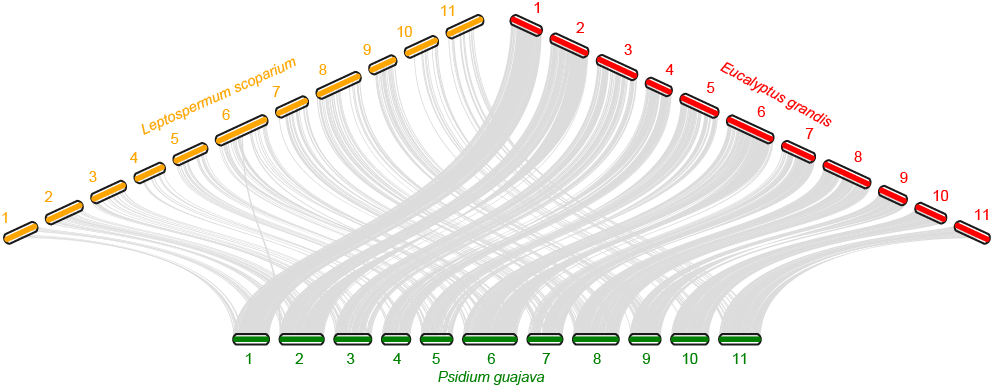
**

**Figure S13.** Syntenic blocks shared between the guava and *L. scoparium* genomes, and between guava and *E. grandis* genomes.

**
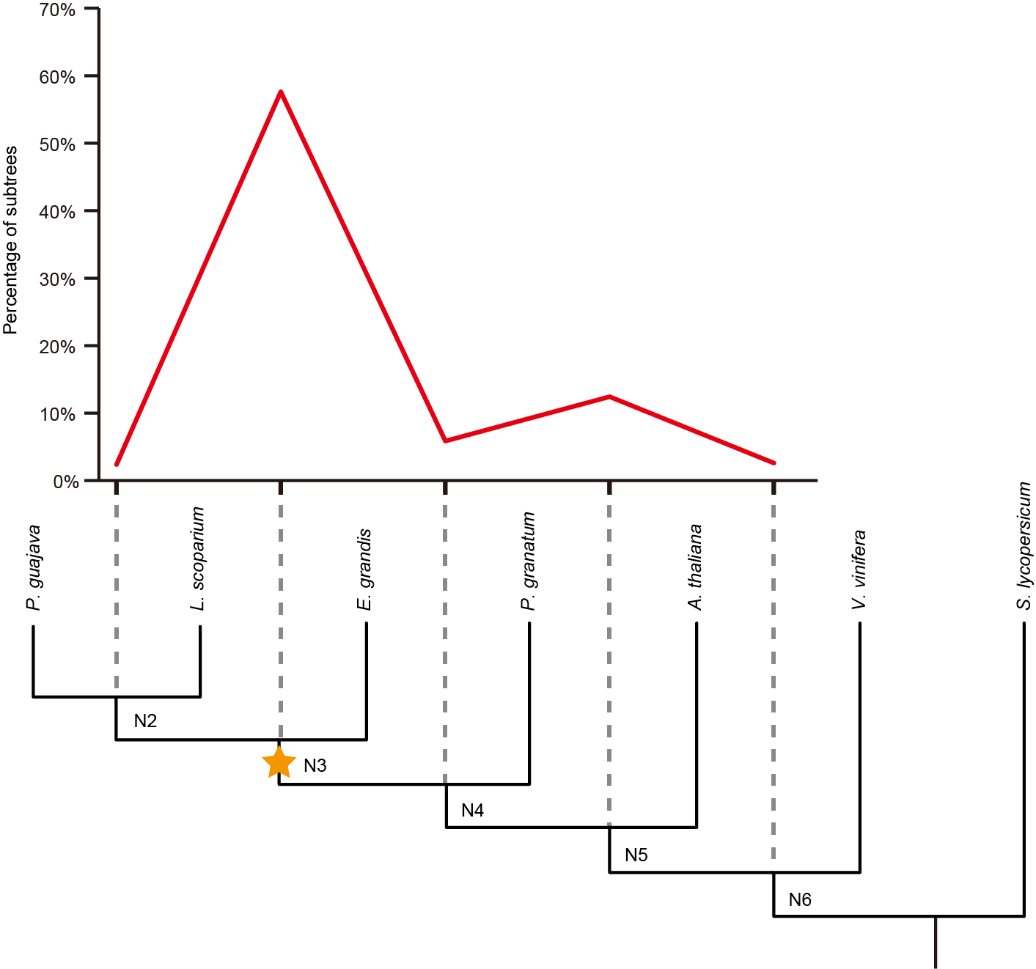
**

**Figure S14.** MAPS result for potential WGDs. Percentage of subtrees indicates percentage of duplicates shared by descendant species at each node. The yellow star represents the WGD event shared by species of Myrtaceae.

**
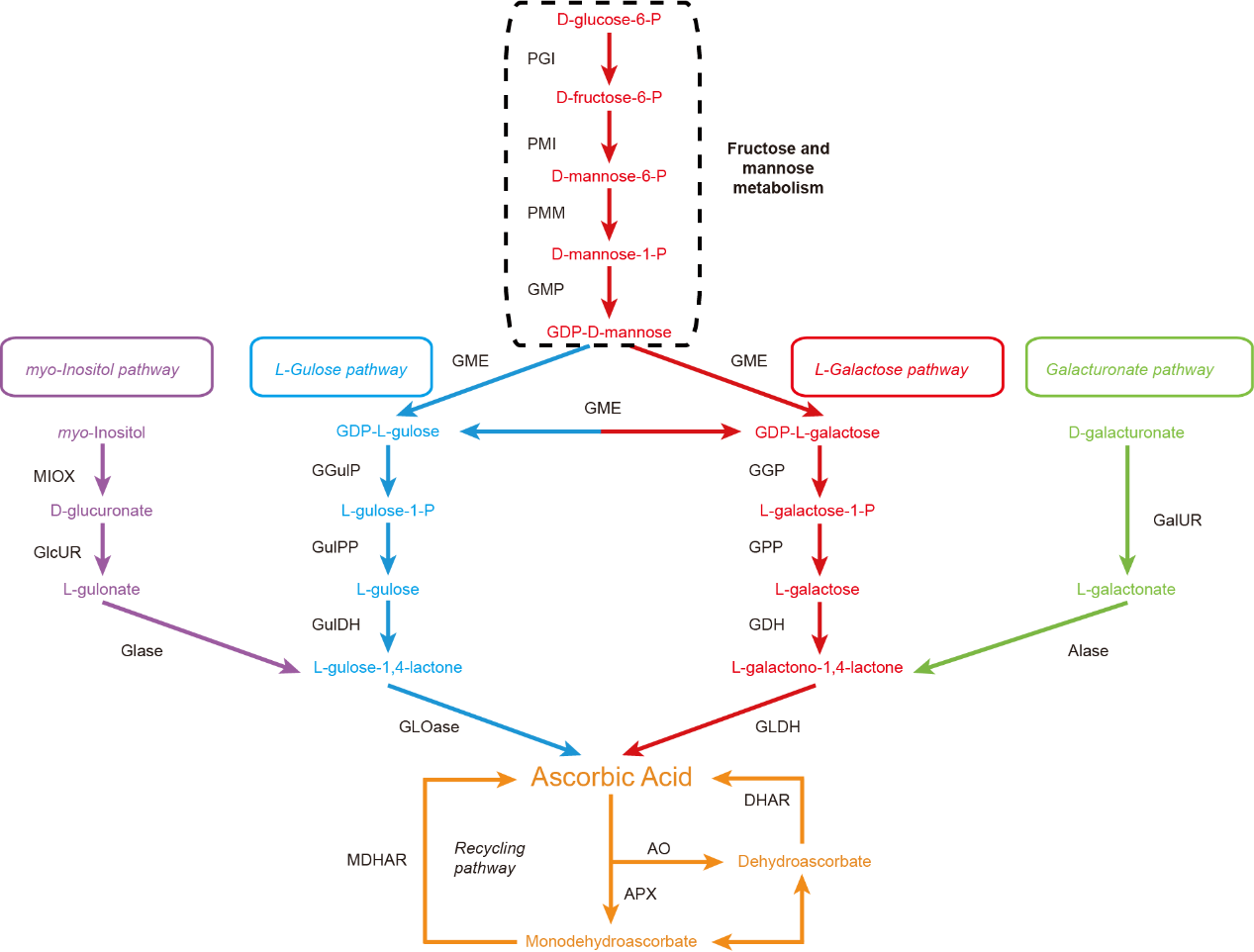
**

**Figure S15.** The four proposed ascorbic acid biosynthesis pathways in higher plants. Gene abbreviations are shown in Table S17.

**
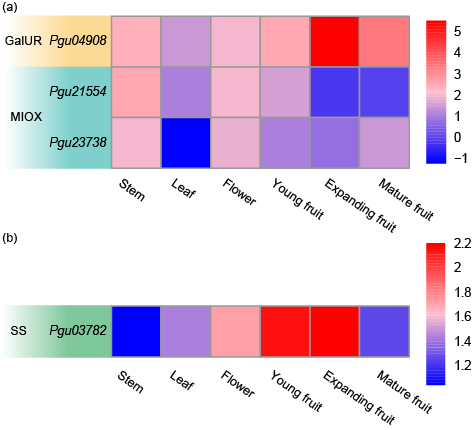
**

**Figure S16.** Heat map of gene transcript abundance in the ascorbic acid biosynthesis pathways (a) and starch biosynthesis (b) in different tissues and at different fruit developmental stages in *P. guajava*. FPKM values are log_2_-based. Red and blue indicate high and low expression levels, respectively. GalUR: D-galacturonate reductase; MIOX: myo-inositol oxygenase; SS: starch synthase.

**
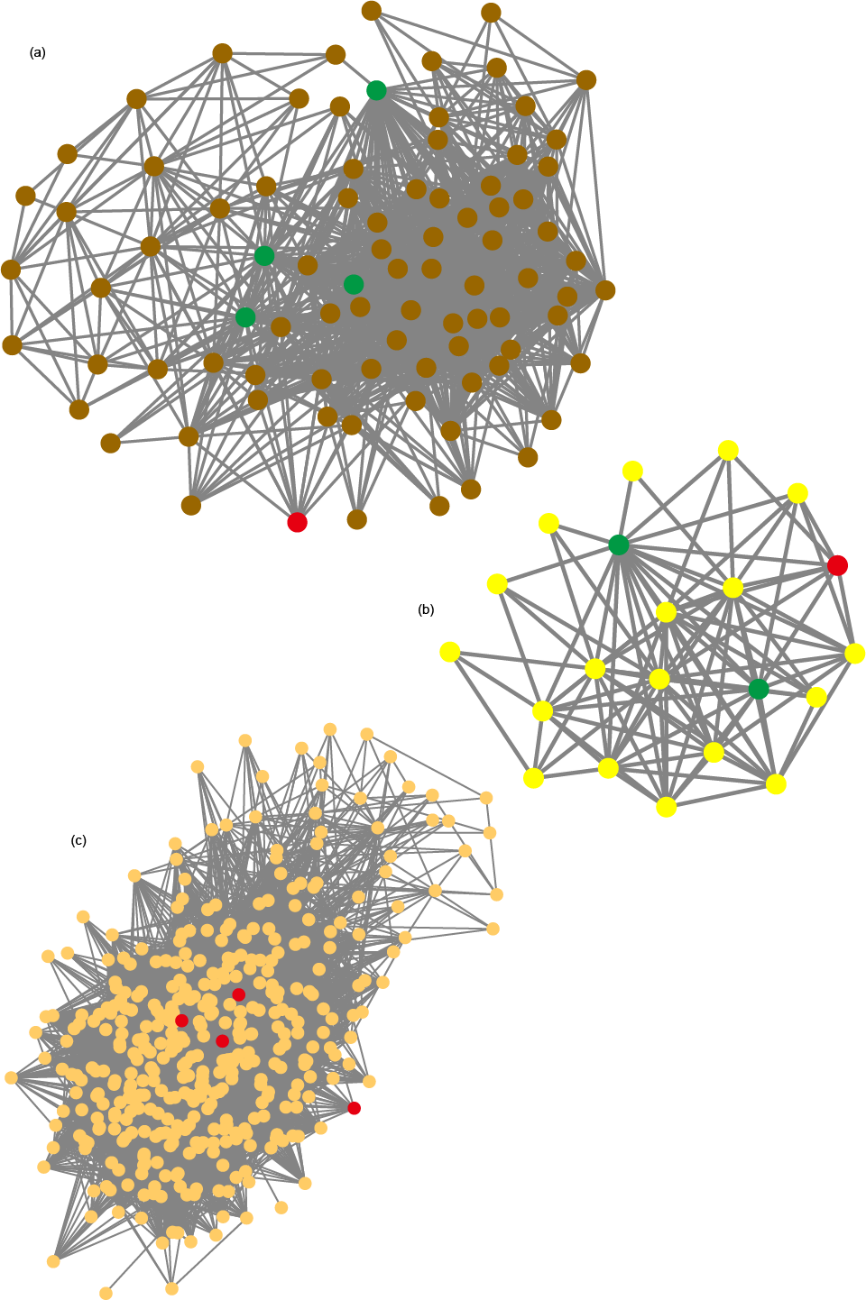
**

**Figure S17.** Gene networks of the tan (a), light yellow (b) and yellow (c) modules. Candidate genes in starch degradation and ascorbic acid biosynthesis pathways are shown in green and red colored circles, respectively. Genes and their abbreviations are shown in Tables S17, S19 and S20.

**
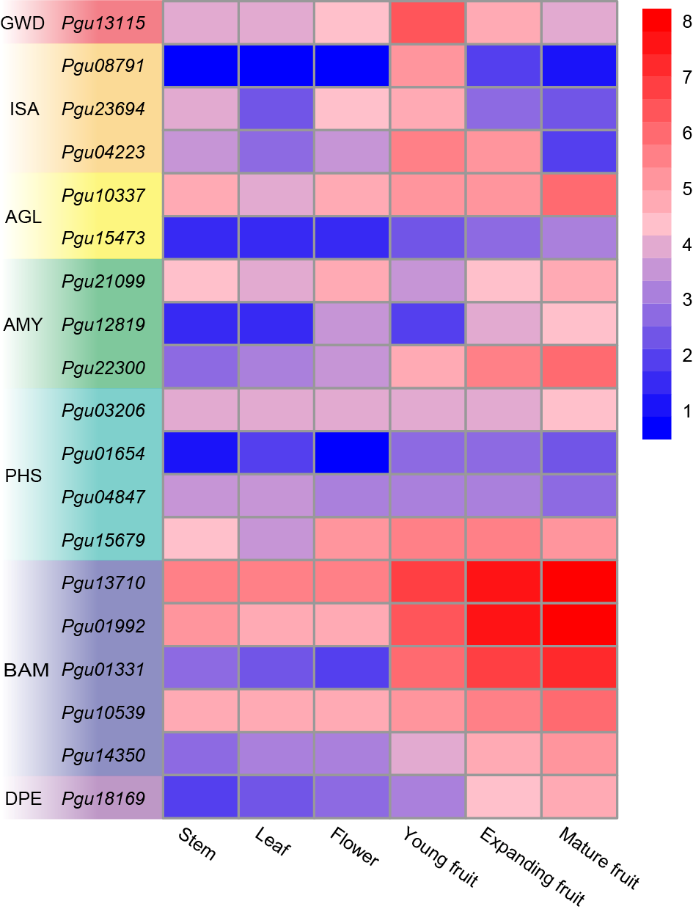
**

**Figure S18.** Heat map of gene transcript abundance in the starch degradation pathway in different tissues and at different fruit developmental stages in *P. guajava*. FPKM values are log_2_-based. Red and blue indicate high and low expression levels, respectively. Gene abbreviations are shown in Table S20.

**
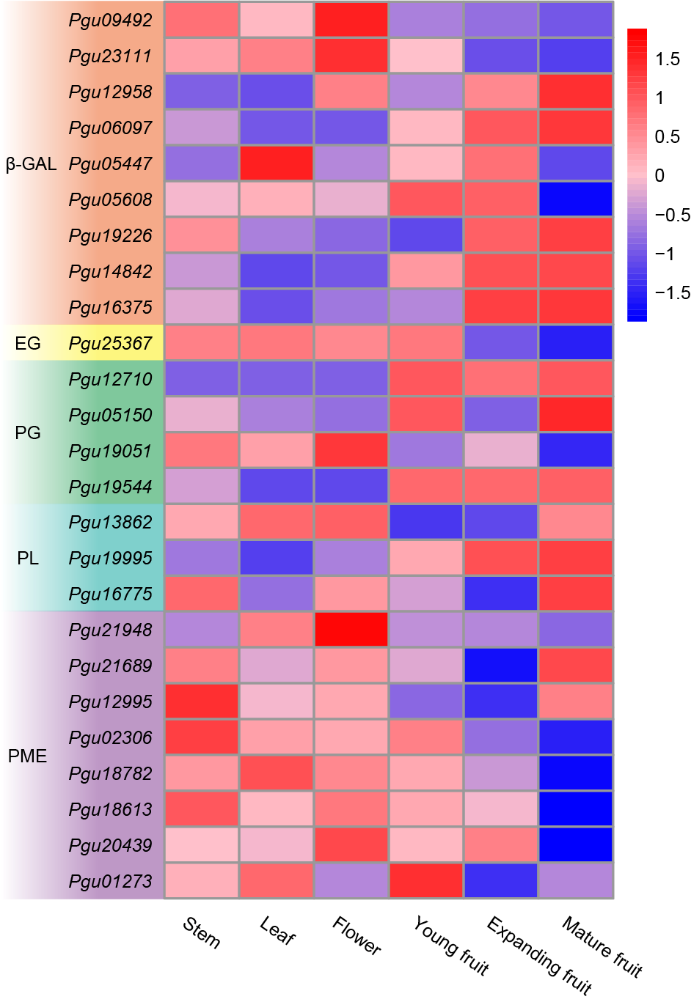
**

**Figure S19.** Heat map of important genes transcript abundance in the cellulose degradation and cell-wall softening pathways in different tissues and at different fruit developmental stages in guava. Gene abbreviations are shown in Table S21.

**
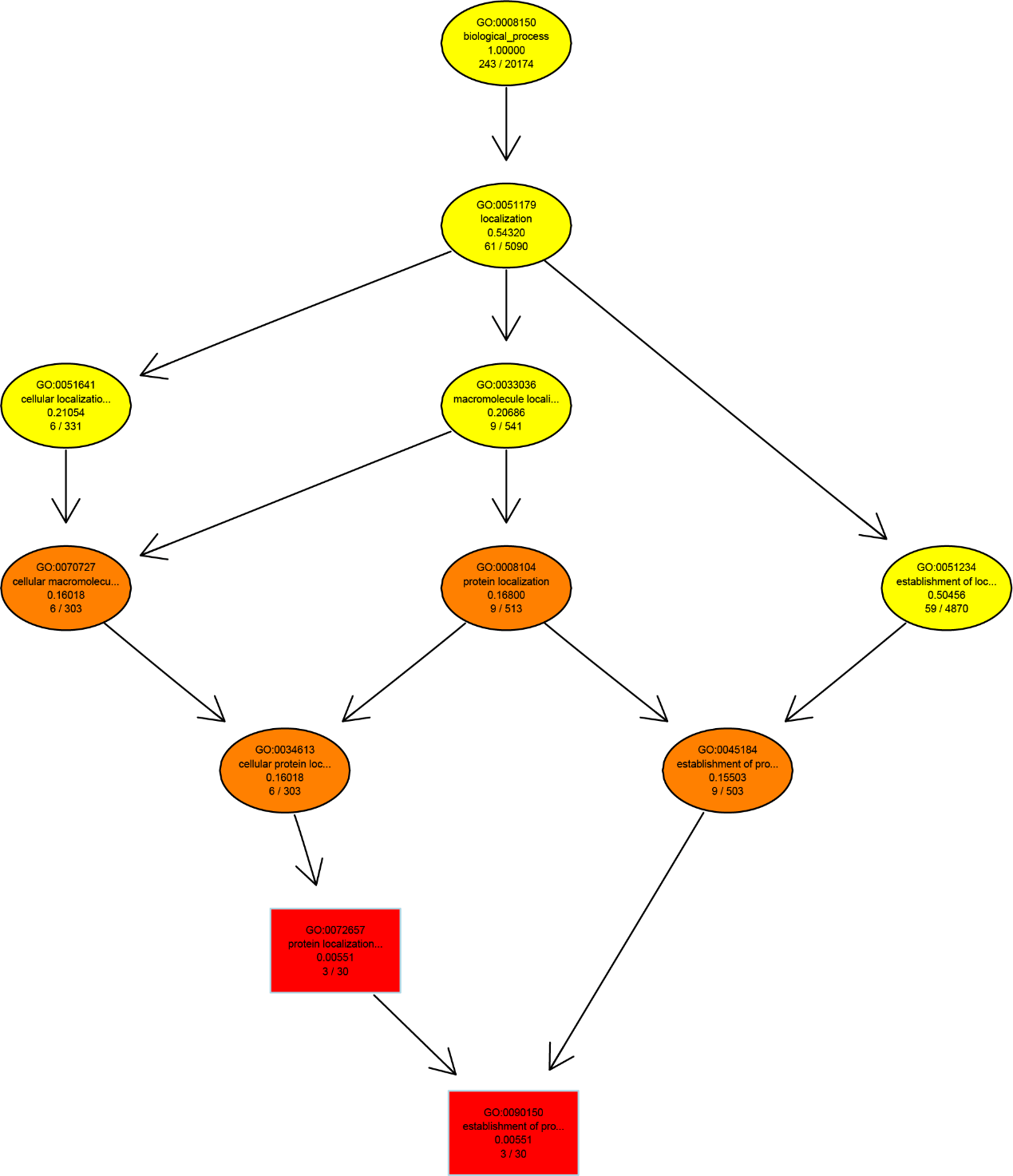
**

**Figure S20.** Gene ontology enrichment of positively selected genes in guava. Directed acyclic graph showed top enriched GO terms belonging to the Biological Process category.

**
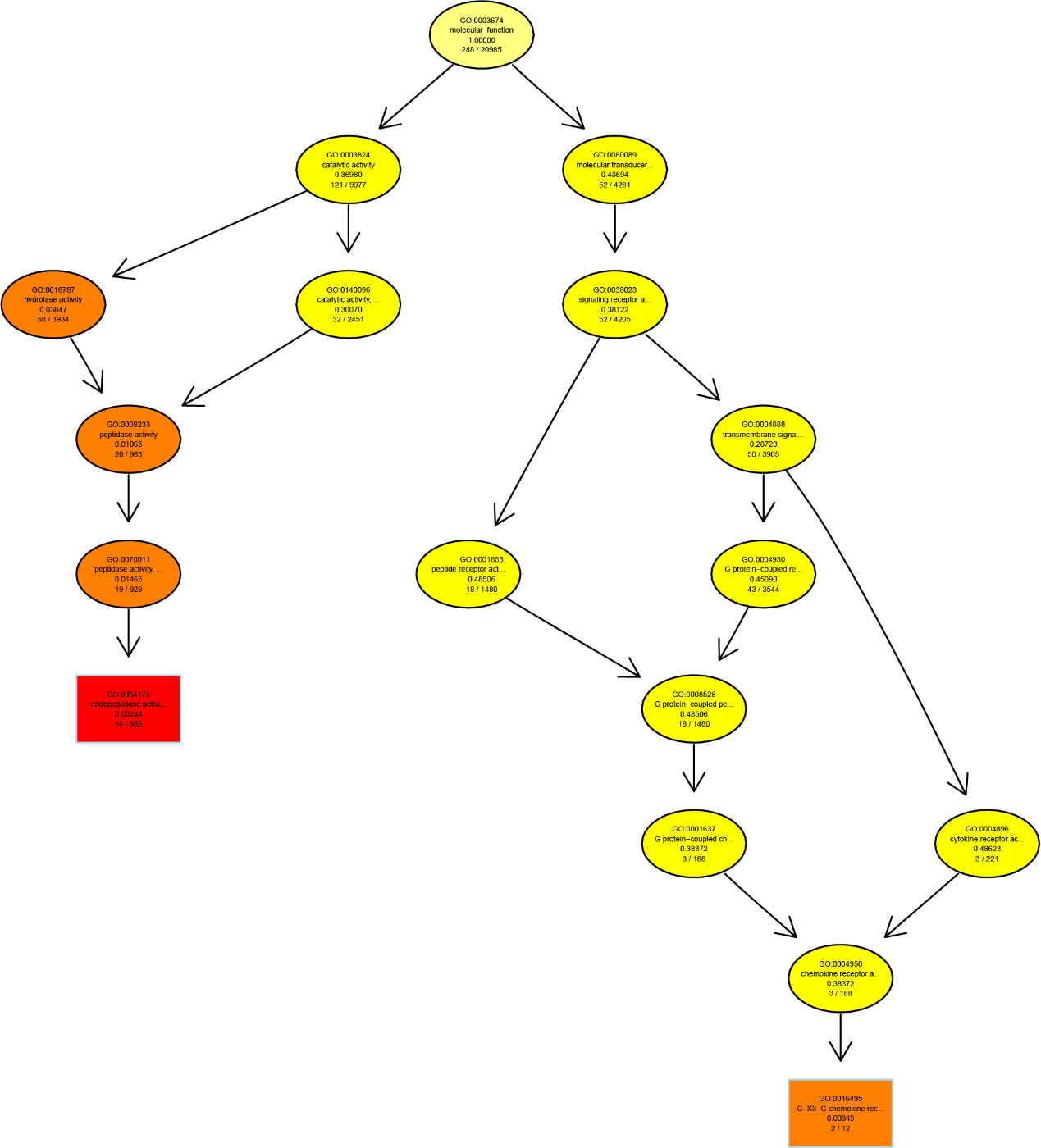
**

**Figure S21.** Gene ontology enrichment of positively selected genes in guava. Directed acyclic graph showed top enriched GO terms belonging to the Molecular Function category.
